# iPSC-Based Modeling of *RAG2* Severe Combined Immunodeficiency Reveals Multiple T Cell Developmental Arrests

**DOI:** 10.1016/j.stemcr.2019.12.010

**Published:** 2020-01-16

**Authors:** Maria Themeli, Amiet Chhatta, Hester Boersma, Henk Jan Prins, Martijn Cordes, Edwin de Wilt, Aïda Shahrabi Farahani, Bart Vandekerckhove, Mirjam van der Burg, Rob C. Hoeben, Frank J.T. Staal, Harald M.M. Mikkers

**Affiliations:** 1 Department of Hematology, Amsterdam UMC, Location VUmc, Cancer Center Amsterdam, Amsterdam 1081 HV, The Netherlands; 2 Department of Immunohematology & Blood Transfusion, Leiden University Medical Center, Leiden 2333 ZA, The Netherlands; 3 Department of Cell & Chemical Biology, Leiden University Medical Center, Leiden 2300 RC, The Netherlands; 4 Department of Clinical Genetics, Leiden University Medical Center, Leiden 2333 ZC, The Netherlands; 5 Department of Clinical Chemistry, Microbiology and Immunology, Ghent University, Gent 9000, Belgium; 6 Department of Immunology, Erasmus Medical Center, Rotterdam 3015 GE, The Netherlands; 7 LUMC hiPSC Hotel, Leiden University Medical Center, Leiden 2333 ZC, The Netherlands

**Keywords:** iPSC, disease modeling, RAG, SCID, CD56^+^CD33^+^, NK cells, T cell development, immunodeficiency

## Abstract

RAG2 severe combined immune deficiency (RAG2-SCID) is a lethal disorder caused by the absence of functional T and B cells due to a differentiation block. Here, we generated induced pluripotent stem cells (iPSCs) from a RAG2-SCID patient to study the nature of the T cell developmental blockade. We observed a strongly reduced capacity to differentiate at every investigated stage of T cell development, from early CD7^−^CD5^−^ to CD4^+^CD8^+^. The impaired differentiation was accompanied by an increase in CD7^−^CD56^+^CD33^+^ natural killer (NK) cell-like cells. T cell receptor D rearrangements were completely absent in *RAG2SCID* cells, whereas the rare T cell receptor B rearrangements were likely the result of illegitimate rearrangements. Repair of *RAG2* restored the capacity to induce T cell receptor rearrangements, normalized T cell development, and corrected the NK cell-like phenotype. In conclusion, we succeeded in generating an iPSC-based RAG2-SCID model, which enabled the identification of previously unrecognized disorder-related T cell developmental roadblocks.

## Highlights

• RAG2-SCID cells show impaired differentiation at several stages of T cell development
• RAG2-SCID T and NK cells fail to undergo legitimate RAG-driven TCR rearrangements
• RAG2-SCID cells exhibit a skewed differentiation toward NK cell-like cells
• RAG2-SCID phenotype is rescued by gene correction

## Graphical Abstract

**Figure:**
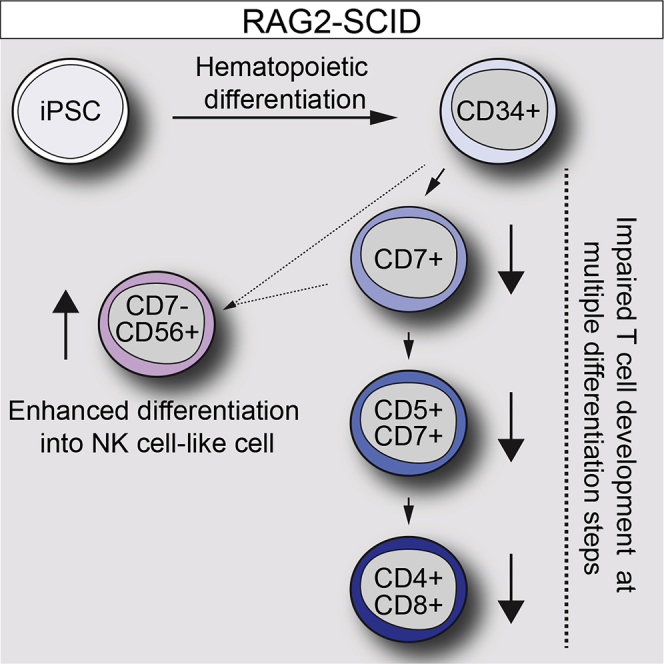

## Teaser

In this article, Mikkers and colleagues model RAG2-SCID using iPSCs and show that the capacity of RAG2-SCID cells to go through T cell development is hampered at multiple transitions from the earliest stage onwards. As a consequence RAG2 mutant cells generate more CD7^−^CD56^+^ CD33+ cells with NK cell properties.

## Introduction

Severe combined immune deficiency (SCID) is a life-threatening disorder caused by a defective acquired immune system due to the absence of functional T cells (Fischer, 2000). In addition to T cells, SCID patients sometimes lack B cells and/or natural killer (NK) cells depending on the underlying genetic mutations. Milder forms of SCID can also occur as a consequence of hypomorphic mutations. One subgroup of SCID patients, accounting for approximately 30% of the SCID cases, lacks both T and B cells (Gaspar et al., 2013). Mutations causing T^neg^B^neg^ SCID most often affect genes that play a role in the rearrangement of the B cell receptor (*BCR*) and T cell receptor (*TCR*) loci, such as recombination activating genes (*RAG*) *RAG1* and *RAG2*, *DCLRE1C*, and *PRKDC* (Dvorak and Cowan, 2010). RAG1 and RAG2 proteins form a tetrameric complex of two RAG1/RAG2 heterodimers, of which one is bound to a 12-recombination signal sequence (RSS) and one to a 23-RSS in the V-D-J regions of the *BCR* and *TCR* loci. The tetrameric RAG complex catalyzes the pairwise cleavage of the RSSs, which are connected through non-homologous end joining, creating the huge V-D-J diversity that underlies the enormously diverse immune repertoire (Notarangelo et al., 2016).

Most of our knowledge of the defects in T cell development in SCID has been gathered from gene-knockout studies in mice. However, the differentiation of human T cells follows a slightly different route compared with that of murine T cells (Blom et al., 1999, Dik et al., 2005, Hao et al., 2008, Weerkamp et al., 2006). To fully understand human SCID, and to study therapeutic interventions, an accessible model that faithfully reflects human SCID is required. Modeling human SCID can be performed *in vitro* by culturing primary CD34^+^ SCID hematopoietic stem/progenitor cells (HS/PCs) on a layer of OP9 cells that express the Notch ligands delta-like 1 (DLL1) or delta-like 4 (DLL4) (Six et al., 2011) or *in vivo* by transplantation of primary long-term repopulating CD34^+^ SCID hematopoietic stem cells (HSCs) into immune-deficient NOD-SCID common γ^−/−^ (NSG) mice (Wiekmeijer et al., 2016). Wiekmeijer and colleagues transplanted HSCs from SCID-X1, IL7R-SCID, and DCLRE1C-SCID patients into NSG animals and observed an earlier block in T cell development than anticipated on the basis of gene expression profiles during human T cell development and corresponding mouse knockouts. This study highlighted that human SCID models are required to investigate the precise underlying developmental defect. However, these experiments fully relied on the availability of primary SCID HS/PCs, which is very restricted due to the rarity of the disorder as well as the wide range of mutations leading to different phenotypes.

Pluripotent stem cells (PSCs) provide a good alternative to model SCID, as human PSCs can be differentiated into T cells *in vitro* (Themeli et al., 2013, Timmermans et al., 2009) and *in vivo* (Galic et al., 2009). Artificial human PSCs can be generated from somatic cells by the overexpression of factors that reset the epigenetic program of somatic cells into that of PSCs (Takahashi et al., 2007, Yu et al., 2007). These so-called induced PSCs (iPSCs) have been successfully used to model SCID-X1 (Menon et al., 2015), JAK3-SCID (Chang et al., 2015), Wiskott-Aldrich syndrome (Laskowski et al., 2016), and RAG1-SCID (Brauer et al., 2016). Since the genetic defect that causes SCID determines the exact stage at which T cell development is stagnated, other SCID disorders should be investigated as well. In addition, the consequences of such a block for the differentiation into other cell types has not been addressed in great detail. Thus it is essential to determine to what extent human iPSC-based SCID models mimic other available SCID models by covering a wide variety of SCID mutations and corresponding phenotypes.

We generated iPSCs from a SCID patient with a homozygous *RAG2* null mutation and demonstrate that *RAG2*-deficient iPSCs have defects throughout every step of T cell development, starting from one of the earliest T-lymphoid-committed stages. The most prominent partial arrests are located at the transition from CD7^+^CD5^−^ cells into CD7^+^CD5^+^ T cells and subsequent differentiation steps. Inhibited T cell differentiation is accompanied by a fate shift toward NK cell-like CD7^−^CD56^+^CD33^+^ cells. Repair of the mutant *RAG2* gene by homologous recombination restored the differentiation phenotype as illustrated by a normal number of CD4^+^CD8^+^ double-positive (DP) T cells with polyclonal TCRδ (TCD) and TCRβ (TCB) rearrangements.

## Results

### Generation of RAG2-SCID iPSCs and Isogenic Control iPSCs

**Figure 1:**
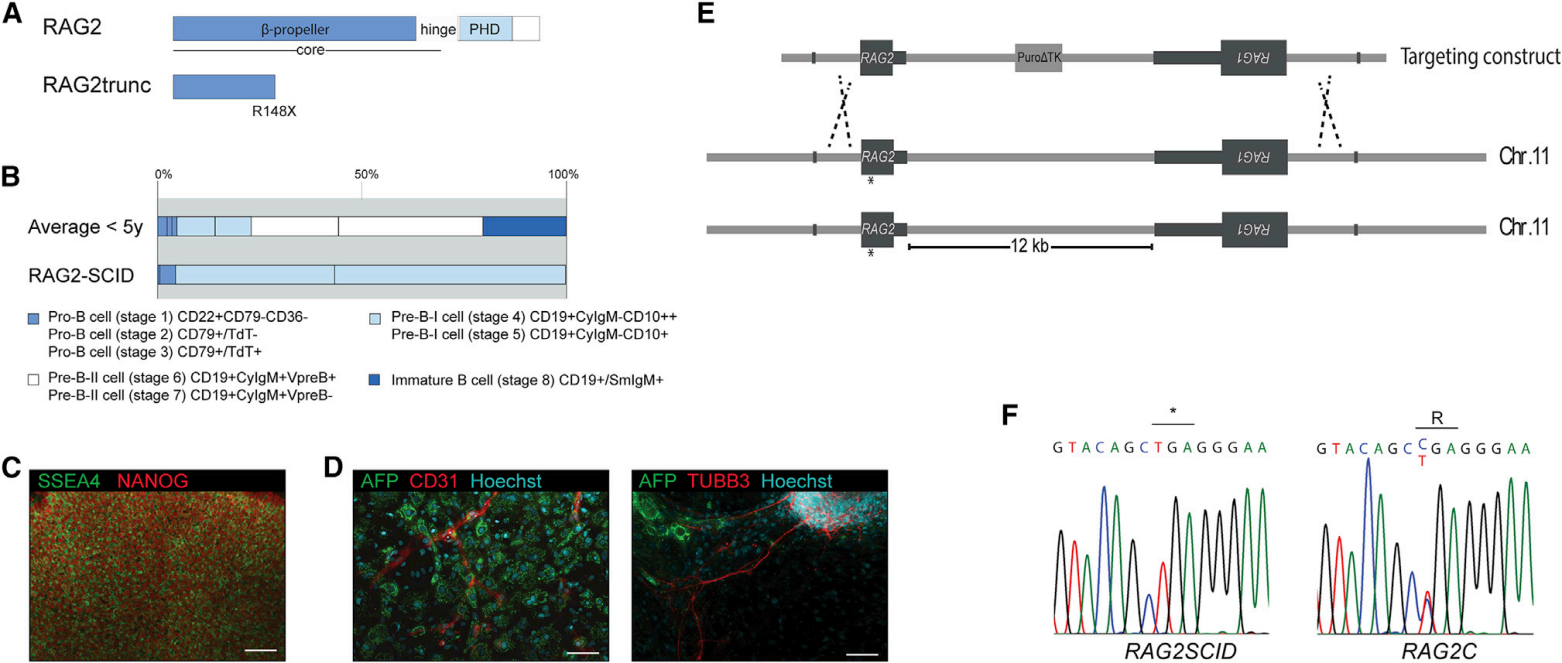
Generation of *RAG2SCID* and *RAG2* Repaired iPSCs (A) Illustration of the mutant RAG2 p.R148X that causes RAG2-SCID. PHD, plant homology domain. (B) Percentages of different B cell populations found in the bone marrow of healthy donors of <5 years (upper bar) and of the RAG2-SCID patient with the homozygous mutation depicted in (A) (lower bar). (C) Fluorescence microscopy analysis of the pluripotency markers NANOG and SSEA4 in one of the generated *RAG2SCID* iPSC clones. (D) Analysis of the capacity of the generated *RAG2SCID* iPSCs to spontaneously differentiate into derivatives of the three germ layers (AFP, endoderm; TUBB3, ectoderm; CD31, mesoderm) by fluorescence microscopy. (E) Schematic illustration of the strategy to repair the *RAG2* mutation using homology-directed repair. (F) *RAG2* sequence analysis of *RAG2SCID* and a repaired *RAG2C* iPSC clone (*RAG2C1*). Scale bars represent 100 μm.

We generated iPSCs from a female RAG2-SCID patient with a homozygous nonsense mutation (p.R148X) in RAG2 (Figure 1A) by transduction of the patient's dermal fibroblasts with a lentiviral vector expressing codon-optimized *OCT4* and *KLF4*, *SOX2*, and *MYC* (Warlich et al., 2011). The selected RAG2-SCID patient demonstrated a complete SCID phenotype indicated by the virtual absence of B and T cells in the peripheral blood (leukocyte count <0.01 × 10^9^/L) and a block in precursor B cell differentiation before the pre-B-II cell stage (Figure 1B). The NK cell count of 0.21 × 10^9^/L was normal, indicative of a T^neg^B^neg^NK^+^ SCID. The generated iPSC clones expressed the pluripotency markers NANOG, OCT3/4, SSEA4, and TRA1-81 (Figures 1C and S1A) and could spontaneously differentiate into the three germ layers (Figures 1D and S1B). We did not identify very large differences in the hemogenic differentiation potential of the different clones upon coculturing with OP9 cells. The percentage of CD31^+^CD34^+^ DP cells ranged from 0.4% to 1.7%, which was lower than with control H1 embryonic stem cells (ESCs) (3.9%) but similar to skin-derived iPSCs from a healthy donor (1.5%). This is in line with the observation that genetic background differences are the major contributors to variations in the differentiation potential of iPSC lines (Kajiwara et al., 2012, Kilpinen et al., 2017). We removed the inserted, single-copy, provirus from one of the RAG2SCID clones through hc.fiber50.FLPe adenoviral vector-mediated FLPe expression to avoid a potential position effect of the integrated provirus (Figures S1D and S1E). Karyotype analysis of the generated iPSC clones did not reveal any gross genomic aberrations (Figure S1I). In addition, the pluripotent potential of the *RAG2SCID* iPSC clones was demonstrated by differentiation into ectodermal, endodermal, and mesodermal derivatives *in vivo* (Figure S1C). Subsequently, the *RAG2* mutation was repaired through conventional homologous recombination (Figures 1E, 1F, S1F, and S1G). To ensure that *RAG1* and *RAG2* transcription fully mirrored *RAG* expression in unmodified, wild-type cells, the intergenic PGKpuromycinΔTK selection cassette was removed from one of the repaired iPSC clones by fiber5.CRE adenoviral vector-mediated CRE recombination (Figure S1H). The pluripotent nature and genomic integrity of the repaired *RAG2* iPSC clones (*RAG2C*) were reconfirmed before further assessment of the phenotype (Figures S1C and S1I).

### Early Hematopoietic Differentiation Is Unaffected in RAG2-SCID iPSCs

**Figure 2:**
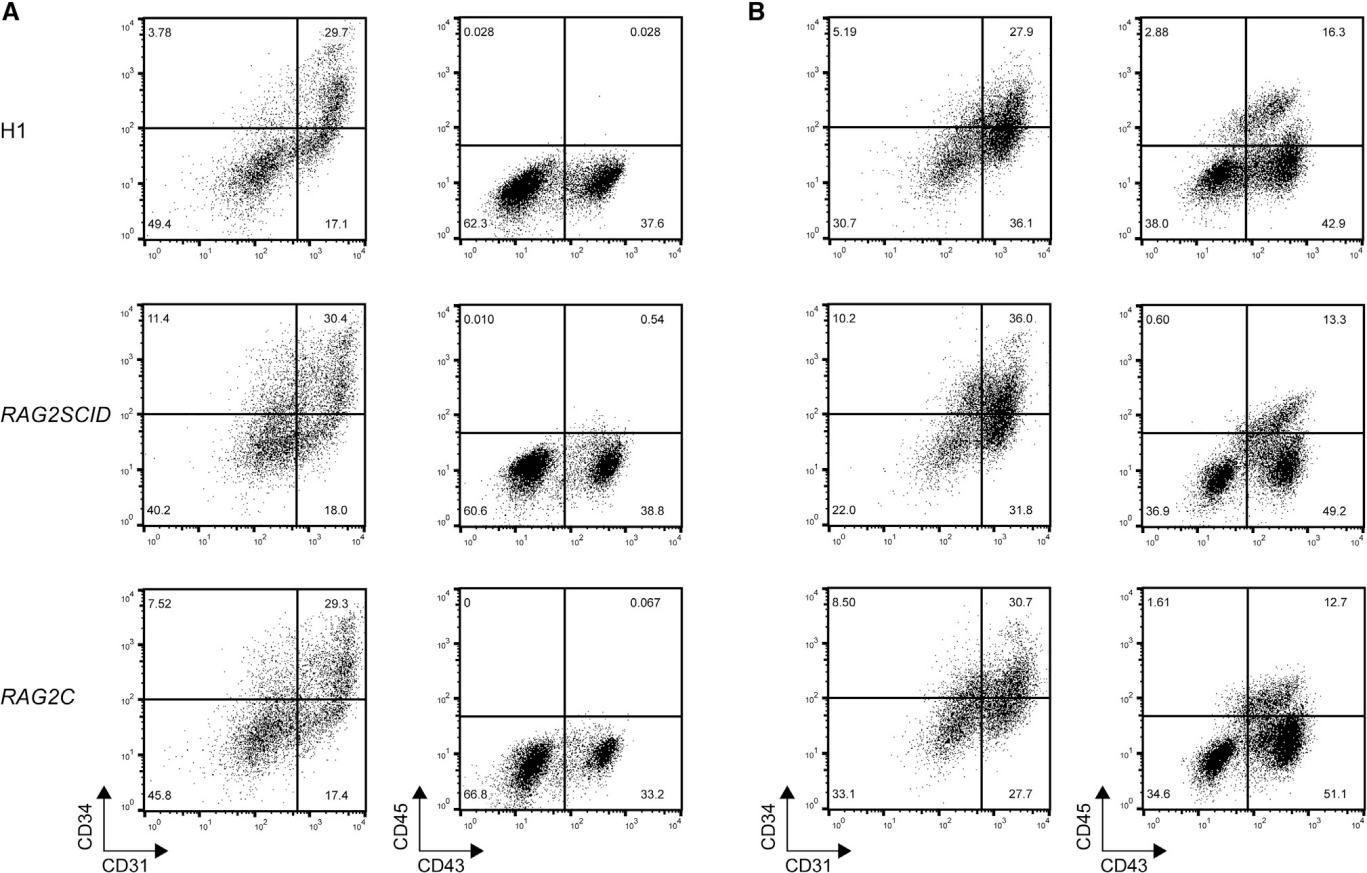
Similar Capacities of *RAG2SCID* and *RAG2C* iPSCs to Differentiate toward Early Hematopoietic Cells *In Vitro* Flow cytometric analysis of hemogenic and hematopoietic cell types (A) at day 9 and (B) at day 12 of *in vitro* hematopoietic differentiation of H1 (top), *RAG2SCID* (middle), and *RAG2C* cells (bottom). One plot is a representative of the results of three independent experiments.

First we wished to compare the capacity of the *RAG2* mutant (*RAG2SCID*) and the *RAG2* repaired (*RAG2C)* iPSC lines to differentiate into early hemogenic endothelial cells and committed hematopoietic progenitors. Early hemogenic colonies from *RAG2SCID* and *RAG2C* were comparable in morphology (Figure S2A). Analysis of the endothelial marker CD31 (Lertkiatmongkol et al., 2016), the hematopoietic and endothelial progenitor marker CD34 (Ditadi and Sturgeon, 2016), the early hematopoietic commitment marker CD43 (Vodyanik et al., 2005), and the universal hematopoietic marker CD45 at day 9 and day 12 of hematopoietic differentiation showed similar hemogenic and committed hematopoietic populations in *RAG2SCID*, *RAG2C*, and control H1 ESCs (Figures 2A, 2B, and S2B).

### RAG2-SCID Cells Are Impaired in T Cell Development

**Figure 3:**
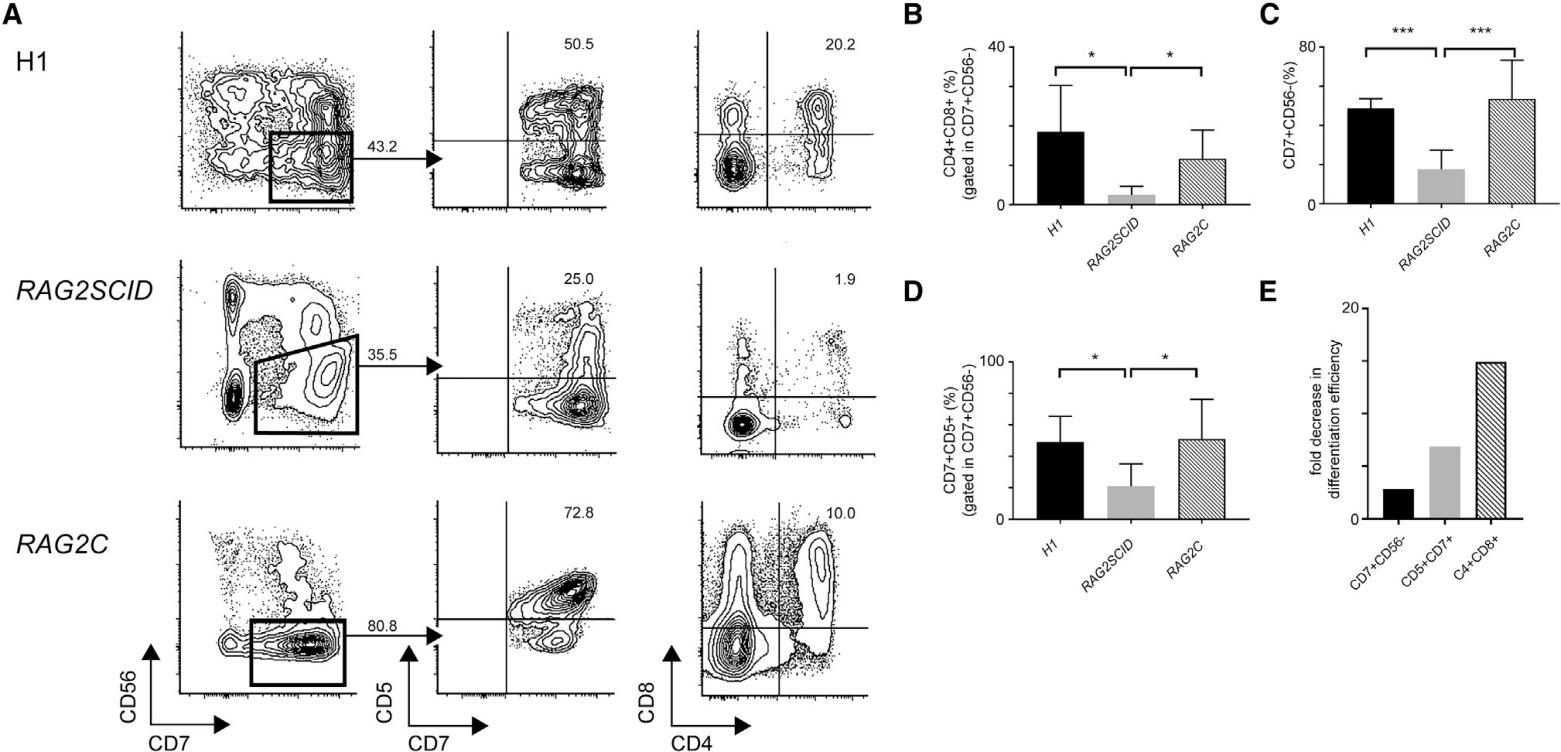
Impaired Differentiation of *RAG2SCID* iPSCs into T Cells *In Vitro* (A) Flow cytometric analysis of the differentiation of H1 (top), *RAG2SCID* (middle), and *RAG2C* (bottom) cells into the T lineage. (B–D) (B) Plotted percentages of generated CD4^+^CD8^+^ DP T cells, (C) early CD7^+^ lymphoid cells, and (D) CD7^+^CD5^+^ cells. (E) Fold decrease in the differentiation of *RAG2SCID* iPSCs into T lineage cells compared with the repaired *RAG2C* iPSCs. Results in (B), (C), (D), and (E) are from three independent experiments using 3 *RAG2SCID* and 2 *RAG2C* clones. Averages + standard deviations are shown. ^∗^p < 0.05, ^∗∗∗^p < 0.001

Since the RAG2 defect likely affects T cell development, we investigated the differentiation of *RAG2SCID* iPSCs into T cells using the OP9-DLL1 coculture system (Themeli et al., 2013). In *Rag2* knockout mice, the T cell differentiation block is located in CD4^-^CD8^-^CD44^-^CD25^+^ (DN3) cells, yielding dramatically reduced numbers of CD4^+^CD8^+^ DP T cells (Shinkai et al., 1992). In the three *RAG2SCID* iPSC clones, CD4^+^CD8^+^ DP T cells were sparsely observed, in contrast to the repaired *RAG2C* iPSC clones and the gold standard H1 ESCs (Figures 3A, 3B, and S3). Immature human T cells are first CD7^+^CD5^−^ and subsequently become CD7^+^CD5^+^ prior to differentiation into immature single-positive (ISP) cells. The CD7^+^CD56^−^ lymphoid population, consisting of CD7^+^CD5^−^ as well as CD7^+^CD5^+^ cells, was significantly reduced in *RAG2SCID* cells, indicating that the *RAG2* mutant cells generated CD7^+^ lymphoid cells less efficiently in comparison with H1 and *RAG2C* cells (Figures 3A, 3C, and S3). Dissection of the CD7^+^ lymphoid population showed that the *RAG2SCID* cells are impaired in becoming CD7^+^CD5^+^ cells and in differentiating further into CD4^+^CD8^+^ DP T cells (Figures 3A, 3D, 3E, and S3). Thus, it appears that *RAG2SCID* cells are hindered in their progression through different stages of T cell development, with the strongest arrest located at the CD7^+^CD5^+^ stage.

### RAG2SCID T and NK Cells Do Not Undergo Legitimate TCR Rearrangements

**Figure 4:**
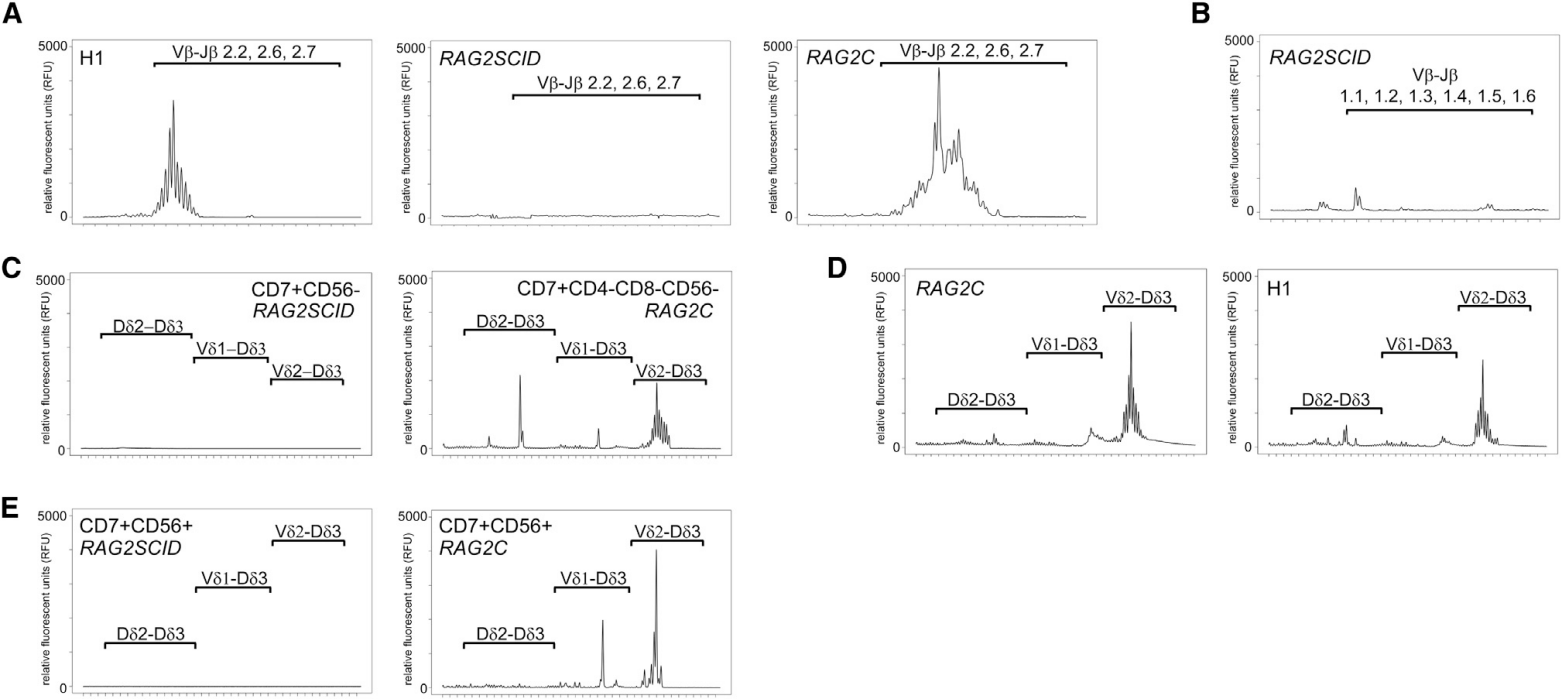
Defective T Cell Receptor Rearrangements in *RAG2SCID* Cells Are Rescued by Repair of *RAG2* (A) GeneScan analysis of TCB (Vβ-Jβ2) rearrangements in whole populations (H1 and *RAG2C*) and in sorted CD7^+^CD56^−^ cells (*RAG2SCID*). (B) GeneScan analysis of TCB (Vβ-Jβ1) rearrangements in sorted CD7^+^CD56^−^ cells (*RAG2SCID*). (C) GeneScan analysis of TCD rearrangements in sorted CD7^+^CD56^−^ cells (*RAG2SCID*) and CD7^+^CD4^−^CD8^−^CD56^−^ cells (*RAG2C*). (D) GeneScan analysis of TCD rearrangements in whole populations (H1 and *RAG2C*). (E) GeneScan analysis of TCD rearrangements in CD7^+^CD56^+^ cells of *RAG2SCID* and *RAG2C*. Fragment length (TCD 150–268 bp and TCB 220–289 bp) is plotted on the x axis. All samples analyzed were from week 4–5 differentiation cultures.

Since RAG2 is, together with RAG1, responsible for the generation of double-strand breaks (DSBs) at the border of an RSS required for the rearrangement of the different TCR segments, we investigated the presence and features of TCB rearrangements by GeneScan analysis (Bruggemann et al., 2004). Whereas H1- and *RAG2C*-derived cells undergo normal TCB rearrangement as shown by the presence of polyclonal T cell populations represented by the Gaussian distribution of Vβ-Jβ2(2.2, 2.6, 2.7) fragment sizes, we could not detect any Vβ-Jβ2 rearrangements in *RAG2SCID* CD7^+^ CD56^−^ cells (Figures 4A and S4A). Nevertheless, the presence of a small fraction of CD4^+^CD8^+^ DP *RAG2SCID* cells in the iPSC-derived T cell differentiation cultures indicates that a few *RAG2SCID* cells could undergo successful positive selection, which is instigated by TCB rearrangements. Therefore, we also investigated the presence of Vβ-Jβ1 rearrangements. We observed very few Vβ-Jβ1 rearrangements in *RAG2SCID* cells (Figure 4B), of which a proportion was of an unexpected size (predicted sizes 240–285 bp). This indicates that the identified rearrangements are rather the result of the repair of rare spontaneous DSBs than of genuine RAG-driven, RRS-based recombination. To further prove the absence of legitimate rearrangements we checked for the presence of immature TCD rearrangements in developing *RAG2SCID* and *RAG2C* T cells. The first rearrangement during T cell development, also for αβ T cells, is the Dδ2-Dδ3 rearrangement in the TCD locus. Dδ2-Dδ3 rearrangements are already observed in very early CD34^+^CD38^−^ thymocytes and peak in CD34^+^CD38^+^ thymocytes (Dik et al., 2005). Dδ2-Dδ3 rearrangements are subsequently followed by immature Vδ2-(Dδ1-Dδ2)-Dδ3 rearrangements. We observed Dδ2-Dδ3 and Vδ2-Dδ3, as well as more mature Vδ1-Dδ3, TCD rearrangements in sorted CD7^+^CD56^−^CD4^−^CD8^−^ *RAG2C* cells but not in sorted CD7^+^CD56^−^ *RAG2SCID* T cells or in *RAG2C* iPSCs (Figures 4C and S4B). Comparison of the TCD rearrangements in unsorted populations of differentiated *RAG2C* cells and H1-derived cells showed very similar Dδ2-Dδ3, Vδ2-Dδ3, and Vδ1-Dδ3 profiles, in terms of both clonality and size distributions (Figure 4D). Of note, Dδ2-Dδ3 rearrangements are more diverse in primary human ISP cells (Figure S4B). Cells that are similar to T cells and are generated in, among others, the thymus are NK cells (Narni-Mancinelli et al., 2011). In particular, CD56^bright^ cells represent an intrathymic NK cell population that is responsive to interleukin-7 (IL-7) (Michaud et al., 2010, Vosshenrich et al., 2006). One NK cell subpopulation, which is thought to have higher fitness than the others, at least in mice, consists of NK cells that express RAG during a certain stage of their development (Karo et al., 2014). As a consequence, a proportion of NK cells, also in men, exhibits TCD rearrangements, in particular immature Vδ2-Dδ3 rearrangements (Fronkova et al., 2005). Since the OP9-DLL1 differentiation protocol used supports the development of CD7^+^CD56^+^ NK cells (Zeng et al., 2017) (Figure 3A), we checked TCD rearrangements in CD7^+^CD56^+^ cells derived from *RAG2SCID* and *RAG2C* iPSCs. Vδ2-Dδ3 rearrangements were prominent in *RAG2C* CD7^+^CD56^+^ cells, confirming the results found in primary human NK cells (Figure 4E). We could also detect Vδ1-Dδ3 rearrangements in *RAG2C* CD7^+^CD56^+^ cells, whereas the most immature Dδ2-Dδ3 rearrangements were largely absent (Figure 4E). In contrast, *RAG2SCID* CD7^+^CD56^+^ cells did not have any TCD rearrangements (Figure 4E). These results highlight that *RAG2SCID* cells are indeed unable to properly recombine the TCR loci in contrast to repaired *RAG2C* cells.

### Differentiation of RAG2-SCID Cells Is Skewed toward NK Cell-like Cells

**Figure 5:**
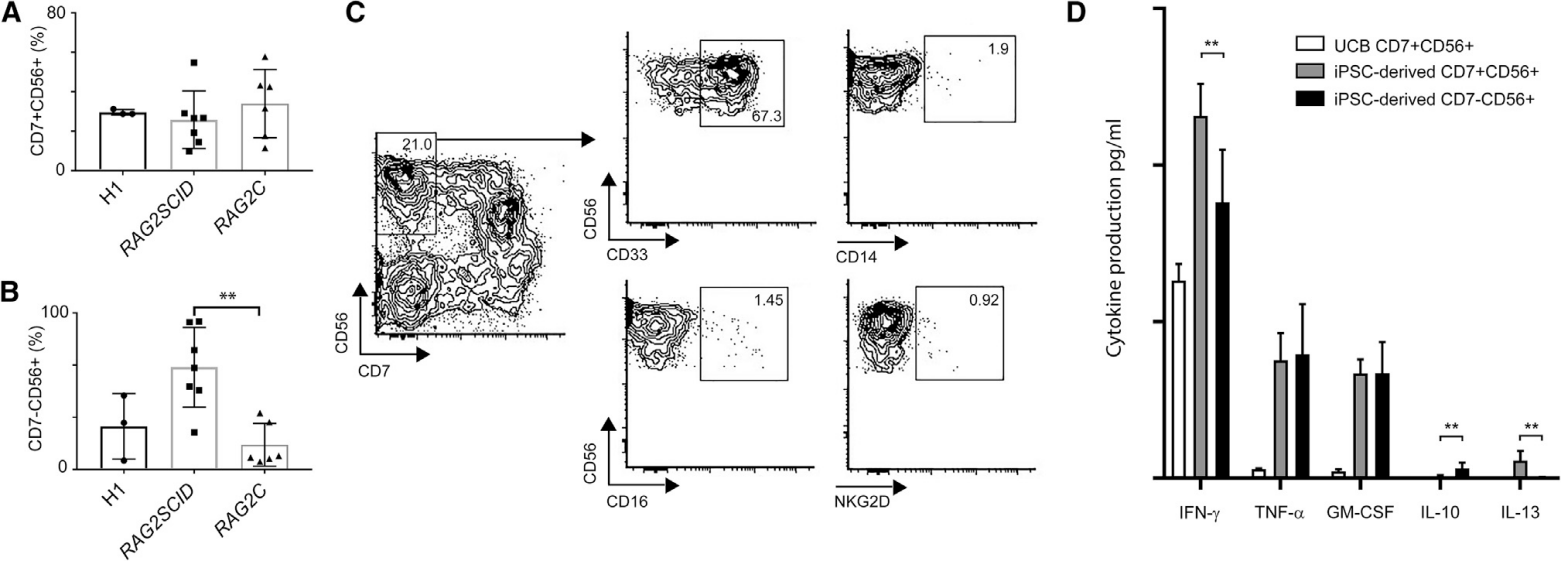
An Increase in CD7^−^CD56^+^CD33^+^ NK Cell-like Cells in *RAG2SCID* iPSC Differentiation Cultures (A and B) (A) Plotted percentages of iPSC-derived CD7^+^CD56^+^ NK cells and (B) CD7^−^CD56^+^ cells. (C) Flow cytometric analysis of CD33 and CD14 (myeloid) and CD16 and NKG2D (NK cell) expression in the CD7^−^CD56^+^ population. (D) Production of cytokines by CD7^−^CD56^+^ and CD7^+^CD56^+^ cells measured after 20 h of stimulation with IL-12, IL-15, and IL-18. Results are from at least three independent experiments. Averages + standard deviations are shown. ^∗∗^p < 0.01.

Next, we wished to investigate whether the reduction in CD7^+^ lymphoid progenitors in *RAG2SCID* cultures was caused by a fate switch of early lymphoid progenitor cells toward cell types that do not require functional TCR rearrangements for their development. Since the OP9-DLL1 differentiation protocol used supports efficient CD56^+^ NK cell development, we zoomed in on the CD56^+^ populations. We did not observe alterations in the percentages of CD7^+^CD56^+^ cells generated from *RAG2SCID*, *RAG2C*, or H1 ESCs (Figure 5A). Instead, we found a significant increase in the generation of *RAG2SCID* CD7^−^CD56^+^ cells (Figure 5B). To our knowledge the only described CD7^−^CD56^+^ cells are monocytic/dendritic cell (DC)-like cells that are positive for CD13, CD33, CD123, and HLA-DR (Milush et al., 2009). We analyzed the CD7^−^CD56^+^ population for the expression of a variety of DC, myeloid cell, and NK cell markers by flow cytometry. The iPSC-derived CD7^−^CD56^+^ population was largely positive for CD33, whereas any other tested monocytic cell, DC, and NK cell markers were absent (Figures 5C, S5A, and S5C). In contrast, the CD7^+^CD56^+^ cells showed a typical NK cell profile, as they expressed CD16, which is commonly found on NK cells, and the NK cell marker NKG2D (Figures S5B and S5C). Since myeloid cell and DC markers were absent, we wondered whether the CD7^−^CD56^+^CD33^+^ cells would respond to NK cell-specific stimuli. CD7^−^CD56^+^ cells stimulated with IL-12, IL-15, and IL-18 produced similar levels of IFN-γ, TNF-α, and GM-CSF compared with iPSC-derived typical CD7^+^CD56^+^ NK cells (Figure 5D). Only the “mutually exclusive” production of IL-10 and IL-13 allowed discrimination of the CD7^−^CD56^+^ and CD7^+^CD56^+^ populations (Figure 5D). In conclusion, our results suggest that inhibition of T cell development by RAG2 deficiency leads to a skewed differentiation toward CD7^−^CD56^+^CD33^+^ NK cell-like cells.

## Discussion

**Figure 6:**
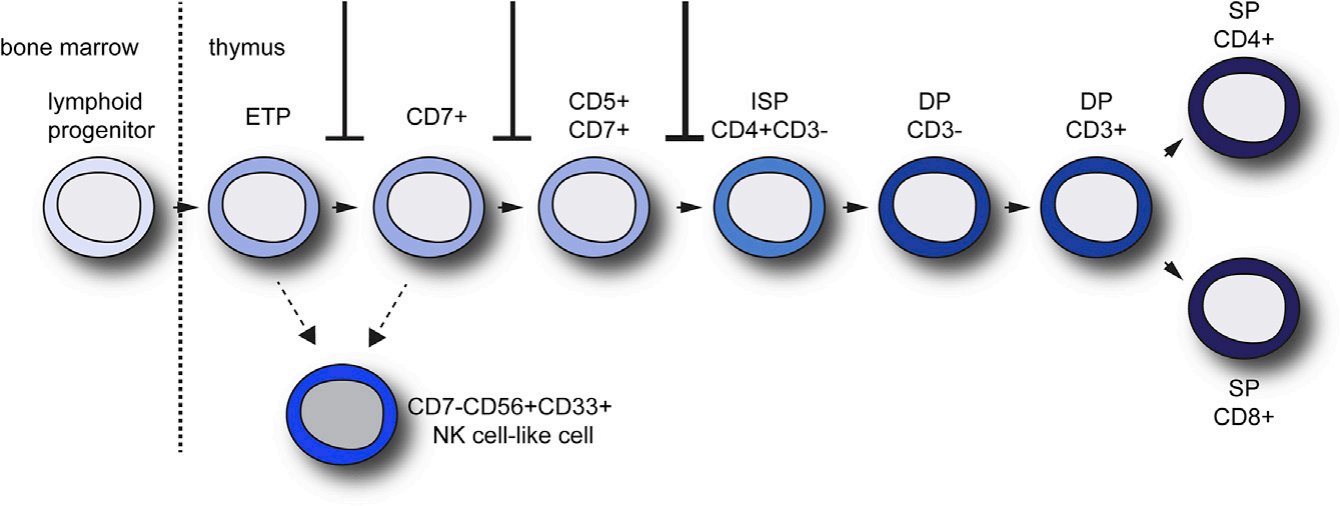
Schematic Illustration of the Impaired T Lineage Differentiation in RAG2-SCID On the basis of our findings we propose partial developmental arrests at several stages of T cell development. The extent of inhibition is represented by line width. As a consequence of the impaired capacity to progress through T cell development, CD7^−^CD56^+^CD33^+^ NK cell-like cells are likely generated from early CD7^−^ or CD7^+^ progenitors.

SCID is caused by a wide variety of mutations and has been mainly investigated using mouse knockout models. It has become evident that human SCID models using mouse knockouts of the affected gene do not represent the human situation very well, likely because human T cell development is different from that in mice (Blom et al., 1999, Dik et al., 2005, Hao et al., 2008, Weerkamp et al., 2006). The best human SCID model to date involves the transplantation of CD34^+^ SCID HS/PCs into NSG animals (Wiekmeijer et al., 2016). However, the availability of CD34^+^ HS/PCs from SCID patients is very limited due to the rarity of the disorder and the wide range of different SCID mutations. Therefore an alternative SCID modeling system is warranted. iPSCs derived from SCID patients have shown promise, but due to the wide variety of SCID mutations and corresponding phenotypes, additional studies are required to evaluate iPSC-based SCID modeling. Using RAG2-SCID iPSCs we observed phenotypic differences with the reported phenotype of *Rag2* knockout mice (Shinkai et al., 1992). *RAG2SCID* iPSC-derived cells showed a partial arrest at every stage of T cell development, ultimately leading to very few CD4^+^CD8^+^ DP T cells (Figure 6). The transitions from CD7^+^CD5^−^ into CD7^+^CD5^+^ cells and from CD7^+^CD5^+^ into CD4^+^CD8^+^ T cells were most profoundly impaired in *RAG2SCID*. Our results are in line with the results obtained from the transplantation of primary bone marrow DCLRE1C-SCID CD34^+^ hematopoietic cells into NSG mice, where a full block at the CD7^+^CD5^+^ stage and a reduced differentiation into CD7^+^CD5^−^ as well as CD7^+^CD5^+^ cells were observed (Wiekmeijer et al., 2016). Artemis, which is encoded by *DCLRE1C*, is involved in opening of the hairpins formed at both nicked RSSs, which are required for V(D)J recombination (Ma et al., 2002). As a consequence the pathogenic phenotypes of RAG-SCID and DCLRE1C-SCID are very similar (Dvorak and Cowan, 2010). Despite the lack of functional Artemis the absence of CD4^+^CD8^+^ DP T cells, TCB and TCD rearrangements were found in the arrested-DCLRE1C-SCID cells, possibly because the hairpins were resolved by a repair mechanism other than Artemis (Wiekmeijer et al., 2016). In contrast, *RAG2SCID* iPSC-derived cells showed only very rare TCB rearrangements, yet immature Dδ2-Dδ3 rearrangements were undetectable. From our data it could be argued that immature TCD rearrangements, which are already detected in the earliest CD34^+^CD38^−^ T progenitor cells (Dik et al., 2005), contribute to a more efficient initiation and progression of T cell development. A genome-wide association study has indeed revealed that a single-nucleotide polymorphism (SNP) within the Dδ2-Dδ3 region influences T cell numbers, particularly of early CD4^−^CD8^−^ thymocytes, highlighting the importance of the region between the Dδ2 and the Dδ3 segments (Clave et al., 2018). Since we did not observe a full block at the CD7^+^CD5^+^ stage, this would indicate that human SCID models based on the coculture of SCID iPSCs and OP9-DLL cells are more lenient. A recent study using RAG1-SCID iPSCs has suggested that multiple T cell waves exist *in vitro* and that only a longer T cell differentiation period of 4–5 weeks better reflects the *in vivo* phenotype (Brauer et al., 2016). In our study we observed very few ISP and DP cells in RAG2-SCID cultures, irrespective of differentiation length. Possibly this difference is explained by the severity of the mutation and/or the affected gene. In addition, the transition from CD3^−^ DP to CD3^+^ DP cells may be affected in RAG2-SCID patients, but this remains to be studied. Nevertheless, we describe and rescue similar blocks at the CD7^+^CD5^−^ and CD7^+^CD5^+^ stages of T cell development, as reported in the RAG1-SCID iPSC-based model.

The absence of TCD rearrangements and the presence of sporadic Vβ-Jβ1 rearrangements, of which a fraction yielded smaller PCR fragment sizes than expected, indicate that the sparse TCB rearrangements found in *RAG2SCID* T lymphoid cells are likely the result of extremely rare random deletions that occurred in only very few cells. These illegitimately rearranged cells are able to bypass the β-selection checkpoint, allowing clonal expansion until the CD4^+^CD8^+^ DP stage. At this stage TCRA needs to rearrange, which is likely to fail in *RAG2* mutant cells, resulting in the lack of mature CD4 and CD8 single-positive (SP) cells. In addition to the T cell phenotype, we observed a strong increase of CD7^−^CD56^+^CD33^+^ cells that do not express other common myeloid lineage, DC, and NK cell markers. However, the observed NK cell-specific cytokine production upon stimulation implies an NK cell-like function. NK cells are generated in multiple tissues, among which is the thymus (Yu et al., 2013). In particular CD56^bright^ cells are believed to represent thymic NK cells (Vosshenrich et al., 2006). Recently, a multicenter study showed that CD56^bright^ NK cells are strongly enriched in the periphery of RAG-SCID patients and that these cells have a rather immature phenotype (Dobbs et al., 2017). It would be interesting to see whether the CD56^bright^ NK cells of RAG-SCID patients encompass a CD7^−^CD56^+^CD33^+^ NK cell-like subset as well. Although the human data contrast with the more mature phenotype of NK cells found in *Rag2*-null mice (Karo et al., 2014), it is obvious that the absence of either *RAG* gene has an effect on the NK cell population. The expression of RAG proteins, somewhere along the route of NK cell differentiation, produces TCD rearrangements in a subset of NK cells. In particular, immature Vδ2-Dδ3 rearrangements are frequently found in NK cells in humans (Fronkova et al., 2005). Likewise, we identified predominantly Vδ2-Dδ3 rearrangements in *RAG2*-corrected CD56^+^CD7^+^ NK cells, but not in *RAG2SCID* NK cells. This reconfirms the rearrangement deficiency in *RAG2SCID* cells and highlights that the applied iPSC-based model system reflects the *in vivo* situation very well.

In conclusion, we present a human RAG2-SCID model that better recapitulates the mutant *RAG2* phenotype than the equivalent mouse knockout, as we could reveal previously unrecognized blocks in T cell development. Although direct comparison with other *in vivo* human SCID modeling systems is still needed, this model likely provides opportunities to link specific mutations to the wide variety of human SCID phenotypes and to evaluate the ability of drugs to rescue the observed phenotype.

## Experimental Procedures

### Generation and Characterization of iPSCs

Passage three fibroblasts from a female RAG2-SCID patient were reprogrammed into iPSCs as described elsewhere (Dambrot et al., 2013). The patient contained a normal number of NK cells in the peripheral blood, whereas B and T cells were <0.01 × 10^9^/L. In the bone marrow there was a complete block prior to the cytoplasmic Igμ-positive pre-B-II stage. In brief: 2 × 10^4^ human fetal fibroblasts were transduced at an efficiency of <10% with the multicistronic lentiviral vector LV.RRL.PPT.SF.hOKSM.idTomato.-preFRT (Warlich et al., 2011) in a 12-well plate. One day after transduction, fresh human fibroblast medium (DMEM/F12, 10% fetal bovine serum [FBS] [Thermo Fisher Scientific], non-essential amino acids [NEAA] [Thermo Fisher Scientific], 100 μM β-mercaptoethanol [Sigma], and penicillin/streptomycin) was added. Cells were harvested using 1× trypsin/EDTA (Thermo Fisher Scientific) 6 days posttransduction, and 1 × 10^4^ cells were seeded onto irradiated CD1 murine embryonic fibroblasts (MEFs) in a 10-cm Petri dish (Greiner) coated with 0.1% gelatin (Sigma) in PSC growth medium (DMEM/F12 [Thermo Fisher Scientific], 20% knockout replacement serum [KRS] [Thermo Fisher Scientific], 10 ng/mL human basic fibroblast growth factor [bFGF] [Peprotech], NEAA [Thermo Fisher Scientific], 100 μM β-mercaptoethanol [Sigma], and penicillin/streptomycin [Thermo Fisher Scientific]). The medium was replaced every other day until the appearance of human ESC-like colonies. iPSC clones were manually picked and further expanded on ESC-qualified Matrigel (Becton Dickinson [BD])-coated 6-well plates in mTESR1 (STEMCELL Technologies). After five passages in mTESR1 the clones were also adapted to feeder conditions and further cultured as described below. For a full description of the characterization protocol we refer to previous studies (Chen et al., 2017, Dambrot et al., 2013). Selected clones were analyzed for the expression of pluripotency markers using immunostaining with primary antibodies recognizing OCT3/4 (1:100; Santa Cruz Biotechnology, cat. no. sc-5279), SSEA4 (1:30; Biolegend, cat. no. 330402), TRA1-81 (1:125; Biolegend, cat. no. 330702), and NANOG (1:500; R&D Systems, cat. no. 963488) and Alexa 488-, Alexa 568-, and Alexa 647-conjugated secondary antibodies (1:500; Thermo Fisher Scientific). Differentiation into three-germ-layer derivatives was assessed by spontaneous differentiation in MEF medium for 3 weeks and subsequent detection of expression of germ-layer-specific markers using immunofluorescence microscopy. Antibodies against AFP (1:25; Quartett, cat. no. 2011200530), CD31 (1:100; Dako, cat. no. M0823), and TUBB3 (1:4,000; Covance, cat. no. MMS-435P) were used. Secondary antibodies were conjugated with either Alexa 488 or Alexa 568 (1:500; Thermo Fisher Scientific). The genome of the clones was analyzed with combined binary ratio labeling (COBRA).

The number of provirus insertions was determined by Southern blot analysis using 10 μg of PaeI-restricted genomic DNA and a NotI/HincII fragment containing the RRE part of the lentiviral reprogramming vector as probe.

To generate teratomas 1 × 10^6^ cells were injected subcutaneously into 8- to 12-week-old immunocompromised female *Rag2*^*−/−*^ mice. Teratomas were isolated 10–16 weeks postinjection. Teratomas were fixed in 4% paraformaldehyde and, subsequently, embedded in paraffin and sectioned. Ectodermal, endodermal, and mesodermal derivatives were visualized by H&E staining and standard light microscopy.

All human materials were collected according to the approval by the Medical Ethics Committee of Erasmus MC (reference no. MEC-2016-606) or LUMC (reference no. P08-087). The experiments involving human materials were done in accordance with the principles outlined in the Declaration of Helsinki. Animal experiments were approved by the Animal Experiments Committee of LUMC (reference no. 12133) and were performed following the recommendations and guidelines set by LUMC and the Dutch Experiments on Animals Act.

### Maintenance Culture of PSCs

*RAG2SCID* iPSC clones (official names: LUMC043iRAG04, LUMC043iRAG05, LUMC043iRAG06, and LUMC043iRAG18), the repaired isogenic controls (LUMC043iRAGC1 and LUMC043iRAGC2), and the wild-type human ESC line H1 (WiCell Research Institute) were cultured on ESC-qualified Matrigel (BD) in mTESR1 (STEMCELL Technologies) or on irradiated MEFs (10,000 cells/cm^2^) (either CD1 [maintenance, genetic modification] or CF1 [T cell differentiation]) on 0.1% gelatin (Sigma)-coated tissue culture plastic in human ESC medium (HESCM) consisting of DMEM/F12 (Gibco), NEAA (Thermo Fisher Scientific), 100 μM β-mercaptoethanol (Sigma), 10 ng/mL human bFGF (Peprotech), penicillin/streptomycin, and 20% KRS (Thermo Fisher Scientific). Cells cultured in mTESR1 were refed once a day and passaged once a week using gentle cell dissociation reagent (GCDR) (STEMCELL Technologies). Cells cultured on feeders were refed once every other day and passaged once a week by cut-and-paste or manual dissociation using either dispase II (Sigma) or collagenase I (Sigma) treatment. For differentiation experiments *RAG2* iPSC lines beyond passage 10 and H1 ESCs beyond passage 27 were used.

### Genomic DNA Isolation

Genomic DNA was extracted from iPSC clones or differentiated cells that were snap frozen as follows: cells were incubated in lysis buffer containing 10 mM Tris-HCl (pH 8.0), 25 mM EDTA (pH 8.0), 0.5% (w/v) SDS, 100 mM NaCl, and 100 μg/mL proteinase K (Thermo Fisher Scientific) at 55°C for at least 5 h, after which the DNA was precipitated with 0.7 volumes of isopropanol. For Southern blotting we included one phenol:chloroform:isoamyl alcohol (25:24:1) (Sigma) extraction step prior to precipitation. The precipitated DNA was washed once with 70% (v/v) ethanol and dissolved in 10 mM Tris-HCl (pH 8).

### Removal of Provirus

LUMC043iRAG18 was selected for removal of the inserted provirus. To this end the iPSC line was first adapted to single-cell passaging as follows. iPSC colonies were dissociated into single cells or small clumps of cells (1–5 cells) by a 1–3 min incubation with TrypLE select (Thermo Fisher Scientific) and replated in 50% fresh HESCM and 50% old HESCM in which the cells were cultured in the presence of 10 μM Fasudil (LC Laboratories). Irradiated CD1 MEFs were seeded on 0.1% gelatin-coated plates 1 day before passaging. Cells were passaged when they were subconfluent, i.e., iPSC colonies almost touching (either next day or after 2 days). The provirus was removed by transduction of single cells in suspension with hcAd.FLPe.F50 adenoviral vector (Gonçalves et al., 2008) at a multiplicity of infection (MOI) of 20 transduction units (TU)/mL. After 1 h at 37°C, single cells were seeded onto MEFs. Removal of the provirus was determined by PCR analysis of the genomic DNA isolated from single-cell-derived clones 11–12 days after transduction.

### Genetic Modification of RAG2SCID iPSCs

RAG2SCID iPSCs were cultured on mTESR1 and Matrigel for the first passages. After passage 5 the iPSCs were cultured on irradiated CD1 MEF feeders (10,000 cells/cm^2^) on 0.1% gelatin (Sigma)-coated tissue culture plastic in HESCM. Prior to electroporation, cells were adapted to single-cell passaging for at least three passages as described above. One day prior to electroporation, cells were passaged in the presence of 10 μM Fasudil (LC Laboratories) to reach a subconfluent density the next day. Single cells (1.5 × 10^7^) were electroporated with 40 μg of PvuI (Fermentas)-linearized *RAG* targeting vector in 800 μL of ice-cold PBS in a 0.4 mL cuvette (Bio-Rad) using a Bio-Rad GenePulser II electroporator (250 V, 500 μF). The targeting vector contained a 28,721 bp *RAG1* and *RAG2* genomic DNA fragment from BAC RP11-669J23 (obtained from the Sanger Institute) that was shuttled into pSuperCOS containing *RAG*-recombineering sequences using pRedET (GeneBridges)-mediated recombineering. To enable selection, a PCR-amplified SbfI-*LoxP.PGKpuroDelTK.LoxP*-SbfI cassette was inserted into the SbfI site in the intragenic region between *RAG1* and *RAG2*, generating a 5′ homology arm of 12,230 bp and a 3′ homology arm of 16,491 bp. Electroporated cells were seeded onto puromycin-resistant MEFs (transduced with a CMV-puro lentiviral vector) in HESCM containing 10 μM Fasudil (LC Laboratories). Cells were selected in HESCM containing 1 μg/mL puromycin (Thermo Fisher Scientific) for 7 days starting 2 days after electroporation. After 11–12 days colonies were numbered, and half of the colony was collected by scraping with a pipet tip from which genomic DNA was isolated. Correctly recombined clones were identified by PCR amplification of the mutated *RAG2* regions using Phusion polymerase (New England Biolabs), followed by PvuII (Fermentas) restriction of the amplified fragments. The PCR fragments were Sanger sequenced. The clones in which one of the mutant *RAG2* alleles was repaired were expanded and officially named LUMC043iRAGC1 and LUMC043iRAGC2. After repair by homologous recombination, LUMC043iRAGC1 was again adapted to single-cell passaging and the selection cassette was removed using CRE recombinase delivery via a first-generation Ad.CRE.F5 vector at an MOI of 50 TU/mL. Cells from which the selection cassette was removed were selected using 200 nM FIAU (Moravek) for 5 days starting 2 days after viral transduction. Removal of the PuroΔTK marker was verified by PCR using intergenic *RAG* and PGKrev primers and genomic DNA control primers (Table S1).

### Hematopoietic Differentiation of iPSCs

The hemogenic potential of the different iPSC clones was determined by coculturing the iPSCs (embryoid bodies [EBs]) on confluent OP9 stroma as described elsewhere (Timmermans et al., 2009). For comparison of the repaired versus parental *RAG2SCID* lines we used the StemDiff hematopoietic kit (STEMCELL Technologies). In brief, we cut iPSC colonies (cultured on hESC-qualified Matrigel [BD] in mTESR1) into small 50–100 μm pieces. These pieces were collected in mTESR1 after short treatment with GCDR (STEMCELL Technologies) and seeded onto Matrigel-coated plates at a density of 4–10 pieces/cm^2^. Medium was changed according the manufacturer's instructions and cells were harvested at day 9 and day 12 of hematopoietic differentiation using TrypLE select (Thermo Fisher Scientific). Differentiation was determined by flow cytometry using anti-CD31 BV605 (BD, no. 562855), anti-CD34 PeRCPe710 (eBioscience, no. 46-0349-42), CD43-FiTC (BD, no. 555475), and anti-CD45 V450 (BD, no. 560368). iPSCs incubated with antibodies and unstained differentiated cells were used as negative controls to determine the gates.

iPSCs were differentiated toward the T lymphoid lineage using a previously described protocol (Themeli et al., 2013). Briefly, undifferentiated iPSC colonies were treated with dispase and transferred to low-attachment plates to allow the formation of EBs in StemPro-34 medium (Thermo Fisher Scientific) supplemented with 2 mM L-glutamine, 1% NEAA, 10 μM β-mercaptoethanol, 100 U/mL penicillin and 100 ng/mL streptomycin, and 50 μg/mL ascorbic acid (Thermo Fisher Scientific). The formation of EBs was facilitated by an overnight incubation in the presence of 30 ng/mL hBMP-4 (R&D Systems [R&D]). EBs were then cultured with hBMP-4 (30 ng/mL) (R&D) and hbFGF (5 ng/ml) (Peprotech) until day 4 to allow for mesoderm induction. Next, hematopoietic specification and expansion were achieved in the presence of hVEGF (20 ng/mL) (Peprotech) and a cocktail of hematopoietic cytokines (hSCF, 100 ng/mL [R&D]; hFlt3L, 20 ng/mL; hIL-3, 20 ng/mL; and hIL-6, 10 ng/mL [all Peprotech]). Day 9 EBs containing hematopoietic progenitor cells were dissociated by treatment with Accutase (Thermo Fisher Scientific) for 20 min, and single cells were then seeded on OP9-DLL1 monolayers to allow for their T lymphoid differentiation in OP9 medium (α-MEM with 20% FBS, 2 mM L-glutamine, 1% NEAA, 10 μM β-mercaptoethanol, 100 U/mL penicillin and 100 ng/mL streptomycin, and 50 μg/mL ascorbic acid [all Thermo Fisher Scientific]) supplemented with SCF, 10 ng/mL (R&D); IL-7, 5 ng/mL (Peprotech); and Flt3L, 10 ng/mL (Peprotech). Once a week cells were harvested and subcultured on OP9-DLL1 monolayers. Medium was replaced once every other day. Differentiation was determined by flow cytometry using the following antibodies: anti-CD1c FiTC, anti-CD4 BV650 (BD, no. 56387), anti-CD5 PE (BD, no. 561897), anti-CD7 PE-Cy7 (eBioscience, no. 25-0079-41), anti-CD8a FiTC (BD, no. 561947), anti-CD11c PerCP-Cy5.5 (BD, no. 565227), anti-CD13 V450 (BD, no. 561157), anti-CD14 APC-Cy7 (BD, no. 557831), anti-CD16 APC-Cy7 (BD, no. 561726), anti-CD33 BV421 (BD, no. 562854), anti-CD43 FiTC (BD, no. 555475), anti-CD45 V450 (BD, no. 560368), anti-CD56 BV785 (Biolegend, no. 362550), CD123 PE (BD, no. 340545), anti-CD141 APC (BD, no. 564123), anti-HLA-DR APC-H7 (BD, no. 561358), anti-NKp46 PE (BD, no. 557991), and anti-NKG2D APC (BD, no. 562064). iPSCs incubated with antibodies and unstained differentiated cells were used as negative controls to determine the gates.

### Statistical Analyses

Statistical analyses were performed using GraphPad Prism software version 7.0. For normal distributions two-tailed Student's t tests were used. A p value of <0.05 was considered statistically significant.

### TCB and TCD GeneScan Analysis

Genomic DNA was directly isolated from frozen sorted and unsorted cells as described above, except the phenol extraction was omitted. Vβ-Jβ rearrangements were amplified using TCRB tube A (Invivoscribe), Amplitaq GOLD (Thermo Fisher Scientific), and Buffer II (Thermo Fisher Scientific) according to the manufacturer's protocol. The TCD rearrangements were detected using a customized primer mix (Invivoscribe) containing 10 pmol of HEX Dδ3-3′ and Vδ1, Vδ2 Dδ2-5′ primers, Amplitaq GOLD (Thermo Fisher Scientific), 2 mM MgCl_2_, dNTP (200 μM each), and Amplitaq GOLD Buffer II (Thermo Fisher Scientific). DNA input was 100 ng and PCR conditions were as follows: 7 min at 95°C and 40 cycles 45 s at 95°C, 45 s at 60°C, 35 s at 72°C. Fragments were separated on a 3730 DNA analyzer (Applied Biosystems), analyzed using the R package Fragman (version 1.0.9), and plotted using R version 3.4.4.

### NK Cell Stimulation

Cells were isolated from umbilical cord blood or iPSC T cell differentiation cultures by fluorescence-activated cell sorting on the basis of CD7 and CD56 expression. Cells were cultured in AIM V medium (no. 31035025, Thermo Fisher Scientific) containing 5% heat-inactivated FBS without or with 10 ng/mL IL-12 (Peprotech), 10 ng/mL IL-15 (R&D), and 20 ng/mL IL-18 (MBL International) in a 96 round-bottom well plate at a density of 12,000 cells/well. After 20 h the supernatants were harvested after spinning the cell suspension at 450 × *g* and snap frozen. Medium without cells served as baseline. Cytokine production was measured with the Bio-Plex Pro Human Cytokine 27-plex Immunoassay (Bio-Rad) according the manufacturer's protocol.

## Author Contributions

M.T. designed and performed experiments; A.C., H.B., H.P., E.d.W., M.C., and A.S.F. performed experiments; M.v.d.B. provided material and advice; and B.V., F.S., and R.H. provided advice. H.M. conceived, supervised, and performed experiments and wrote the manuscript with support from M.T., R.C.H., and F.S.

## Supplemental Information

Document S1. Figures S1–S5 and Table S1

Document S2. Article plus Supplemental Information
