# Supplementary material for: iPSC-Based Modeling of RAG2 Severe Combined Immunodeficiency Reveals Multiple T Cell Developmental Arrests
Source: Stem Cell Reports. 2020 Jan 16;14(2):300–11. doi: 10.1016/j.stemcr.2019.12.010 (PMC7013232; doi:10.1016/j.stemcr.2019.12.010)
Supplement: Document S1. Figures S1–S5 and Table S1 [file mmc1.pdf]

Stem Cell Reports, Volume *14*

Supplemental Information

iPSC-Based Modeling of *RAG2* Severe Combined Immunodeficiency Reveals Multiple T Cell Developmental Arrests

Maria Themeli, Amiet Chhatta, Hester Boersma, Henk Jan Prins, Martijn Cordes, Edwin de Wilt, Aïda Shahrabi Farahani, Bart Vandekerckhove, Mirjam van der Burg, Rob C. Hoeben, Frank J.T. Staal, and Harald M.M. Mikkers

Inventory of Supplemental Information

Themeli et al.

Figure S1: iPSC characterization data that are not presented in Figure 1.

Figure S2: Hematopoietic differentiation data that are not incorporated into Figure 2.

Figure S3: T cell differentiation data of all (3 *RAG2SCID* and 2 *RAGC*) clones analysed.

Figure S4: TCR rearrangement data additional to the results shown in Figure 4.

Figure S5: Expression analysis of monocytic, DC, and NK cell markers in the CD56+ populations from *RAG2SCID* and *RAG2C* that are not presented in Figure 5. In addition, the marker profile of the CD56+ cells from umbilical cord blood that served as positive control in the stimulation experiment is depicted.

Figure S1

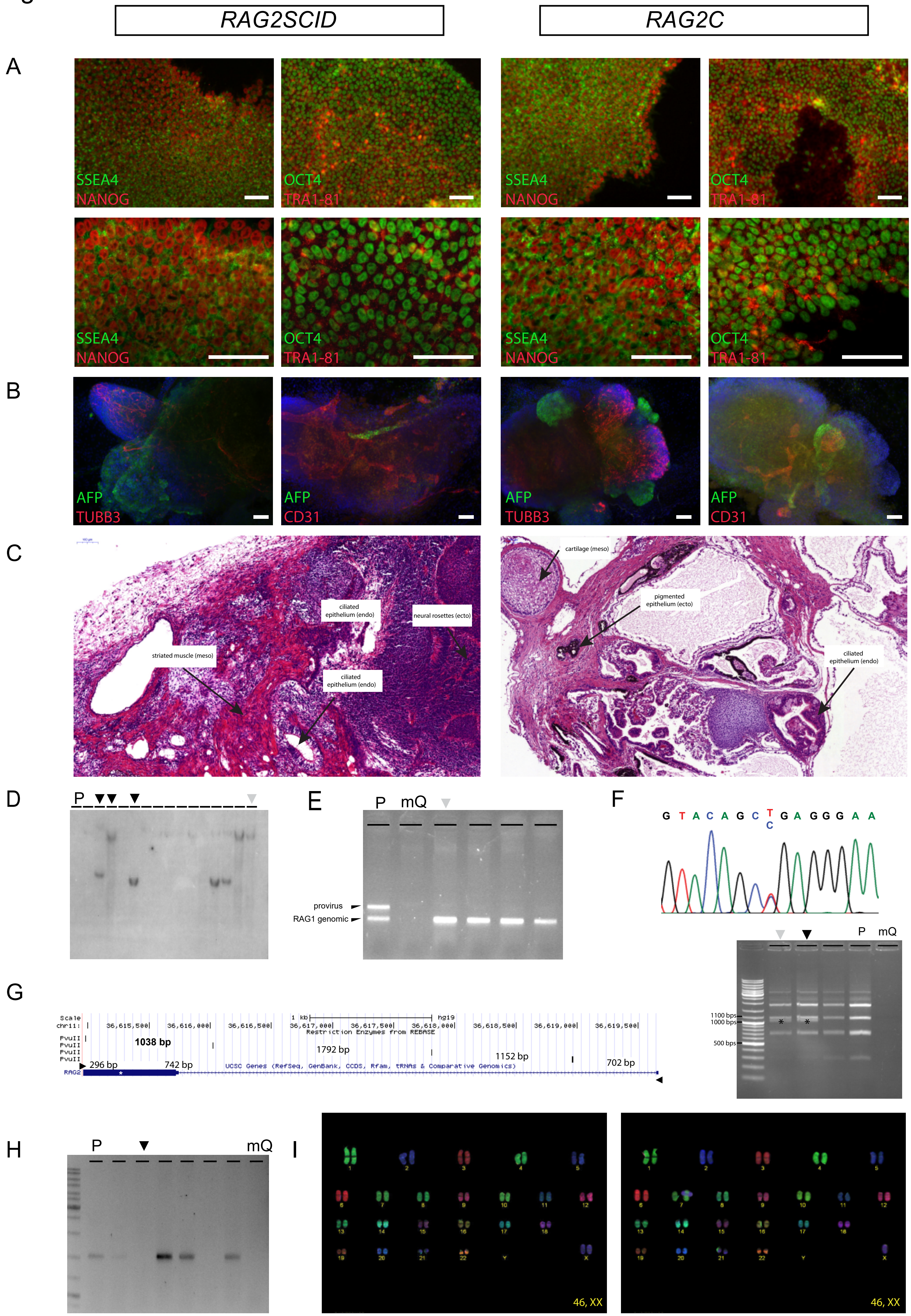
RAG2SCID
RAG2C
A
SSEA4
NANOG
OCT4
TRA1-81
B
AFP
TUBB3
CD31
C
striated muscle (meso)
ciliated epithelium (endo)
neural rosettes (ecto)
cartilage (meso)
pigmented epithelium (ecto)
D
P
E
P mQ
provirus
RAG1 genomic
F
G T A C A G C T G A G G G A A
C
P mQ
1100 bps
1000 bps
500 bps
G
Scale
chr11:
36,615,500
36,616,000
36,616,500
1 kb
36,617,000
36,617,500
36,618,000
hg19
36,618,500
36,619,000
36,619,500
Restriction Enzymes from REBASE
PvuII
PvuII
PvuII
PvuII
1038 bp
1792 bp
1152 bp
702 bp
296 bp
742 bp
UCSC Genes (RefSeq, GenBank, CCDS, Rfam, tRNAs & Comparative Genomics)
RAG2
H
P
mQ
I
46, XX
46, XX

Figure S1. Characterization of generated *RAG2SCID* and repaired *RAG2C* iPSC. A. Immunofluorescence of the pluripotent stem cell markers SSEA4, NANOG, OCT4 and TRA1-81. B. Immunofluorescence of the spontaneous differentiation into three germ layers in vitro. AFP (endoderm), TUBB3 (ectoderm), CD31 (mesoderm). C. HE staining of teratomas. Structures of the three germ layers are indicated by arrows. D. Provirus insertion analysis of multiple *RAG2SCID* clones. Arrows indicate clones used in the differentiation experiments. E. PCR to assess removal of the provirus from one *RAG2SCID* clone (gray arrow in D) by Fib50.FLPe treatment. F. DNA sequencing chromatogram of *RAG2C2* indicating repair of the nonsense mutation in one of the mutant alleles. G. UCSC genome browser (hg19) view depicting the PvuII fragments of the amplified *RAG2* region (left). The nonsense mutation (*) in *RAG2* creates an additional PvuII restriction site in the 1038 bps fragment yielding one 296 bps and one 742 bps fragment. PvuII restriction of the amplified *RAG2* fragment shows an extra 1038 bps fragment (indicated by black asterisk) in the repaired clones (*RAG2C1* (grey arrow) and *RAG2C2* (black arrow). P= one of the parental *RAG2SCID* clones. H. PCR analysis of the selection cassette. Black arrow represents clone *RAG2C1* from which the selection cassette was removed. P= the parental clone. H. Karyotype analysis using COBRA-FISH. *RAG2SCID* (left), *RAG2C* (right). One of the X-chromosomes (*RAG2C*) is located on top of one of the chromosomes 7 in this spread. Scale bars represent 100 μM.

Figure S2

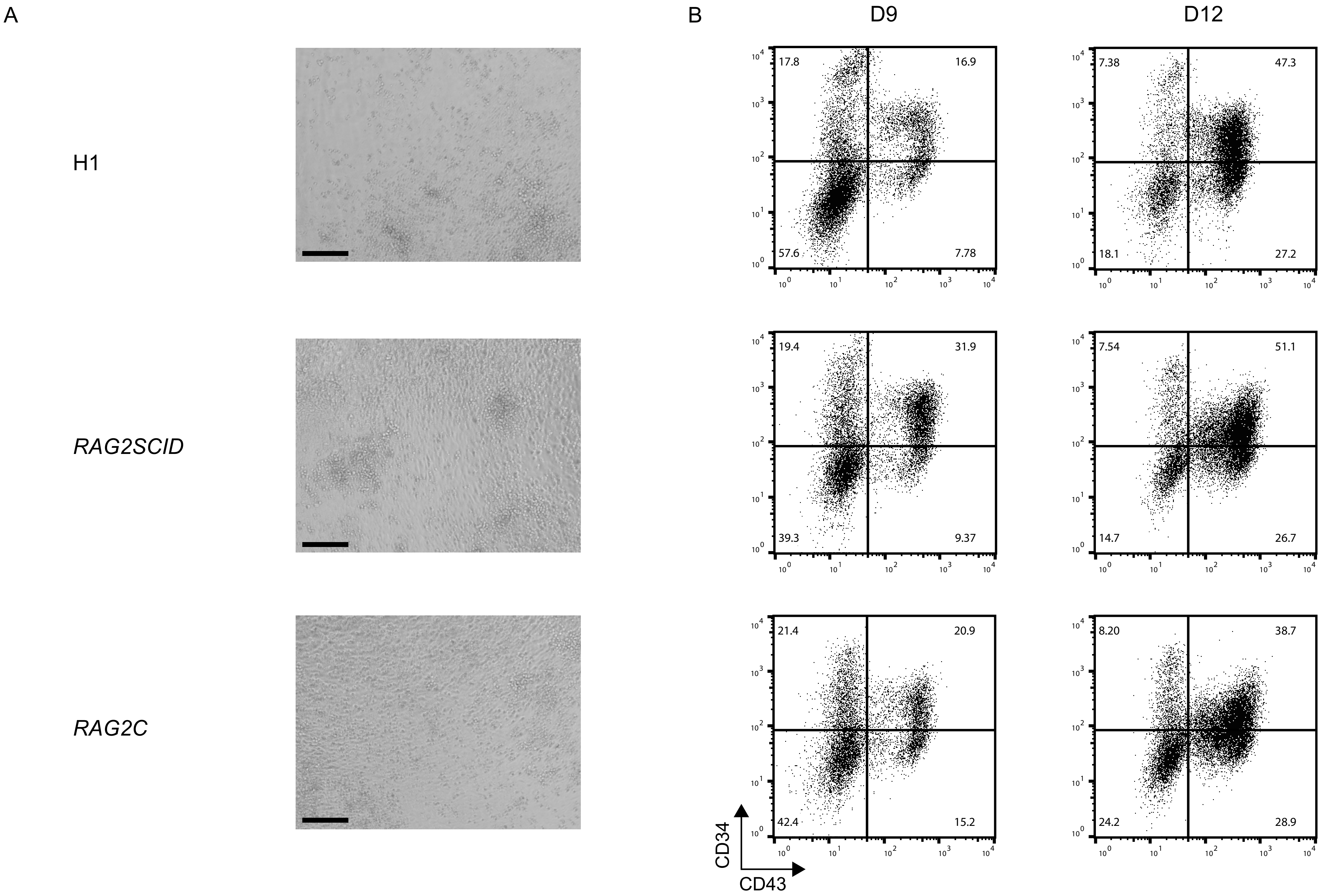

Figure S2. Early hematopoietic differentiation RAG2SCID and repaired RAG2C iPSC is very similar. A. Bright field images of hematopoietic differentiation cultures at day 9 of differentiation. B. Flow cytometric analysis of CD34 and CD43 in hematopoietic differentiation cultures at day 9 (left) and day 12 (right) of differentiation. H1 (upper panel), *RAG2SCID* (middle panel), and *RAG2C* (lower panel). Scale bar represents 200 μM. Plots are a representative example from three independent experiments.

Figure S3

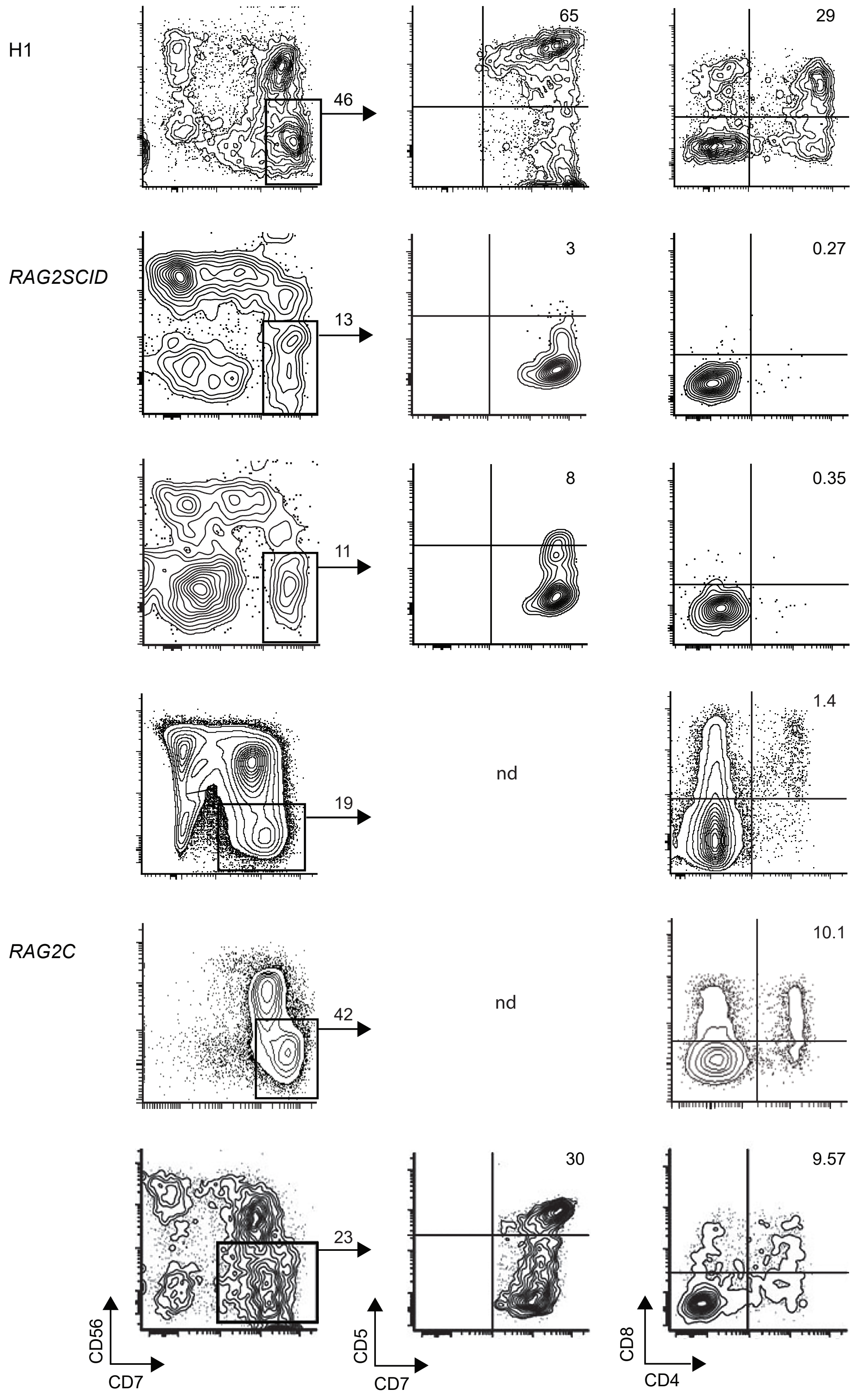

Figure S3. T cell differentiation and rescue across multiple mutant and repaired RAG2SCID iPSC clones. Additional representative flow cytometry plots showing the differentiation into the T lineage of H1 ESCs, three *RAG2SCID* iPSC clones and two repaired isogenic *RAG2C* iPSC clones. nd = not done

Figure S4

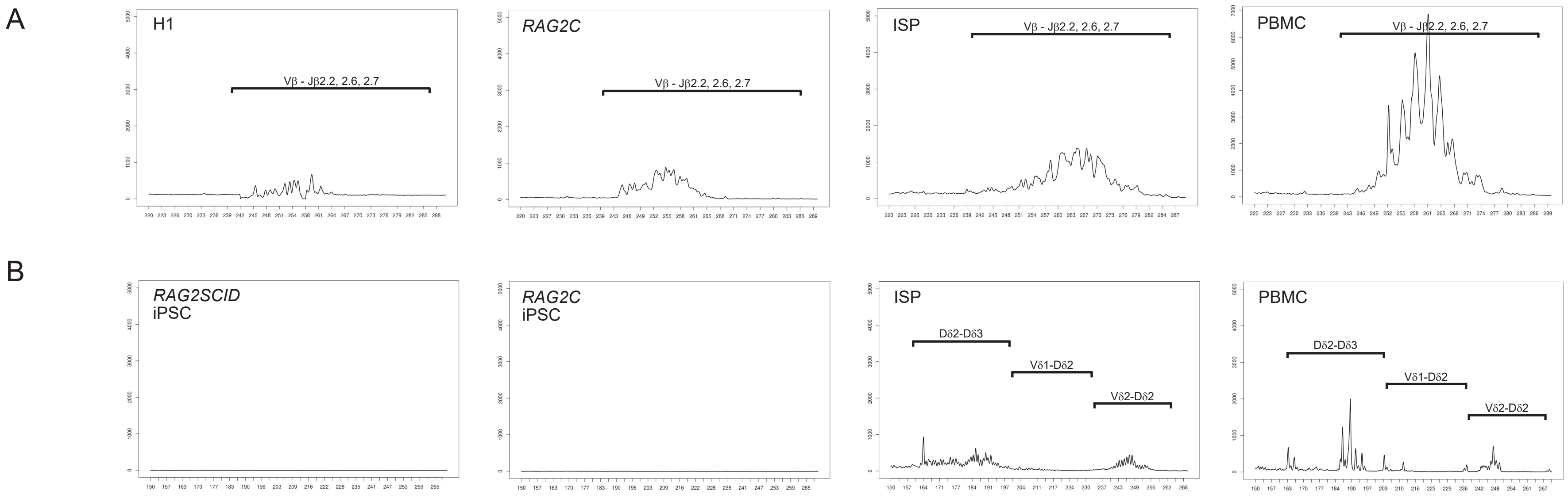

Figure S4. TCR rearrangements in repaired RAG2SCID T cells.
A. GeneScan analysis of TCB (Vβ-Jβ2) rearrangements in CD4+CD8+ sorted H1 and RAG2C cells, as well as in primary human ISP cells and peripheral blood mononuclear cells (PBMC). B. GeneScan rearrangements of early TCD rearrangements in RAG2SCID and RAG2C iPSC, primary ISP cells and PBMC.

Figure S5

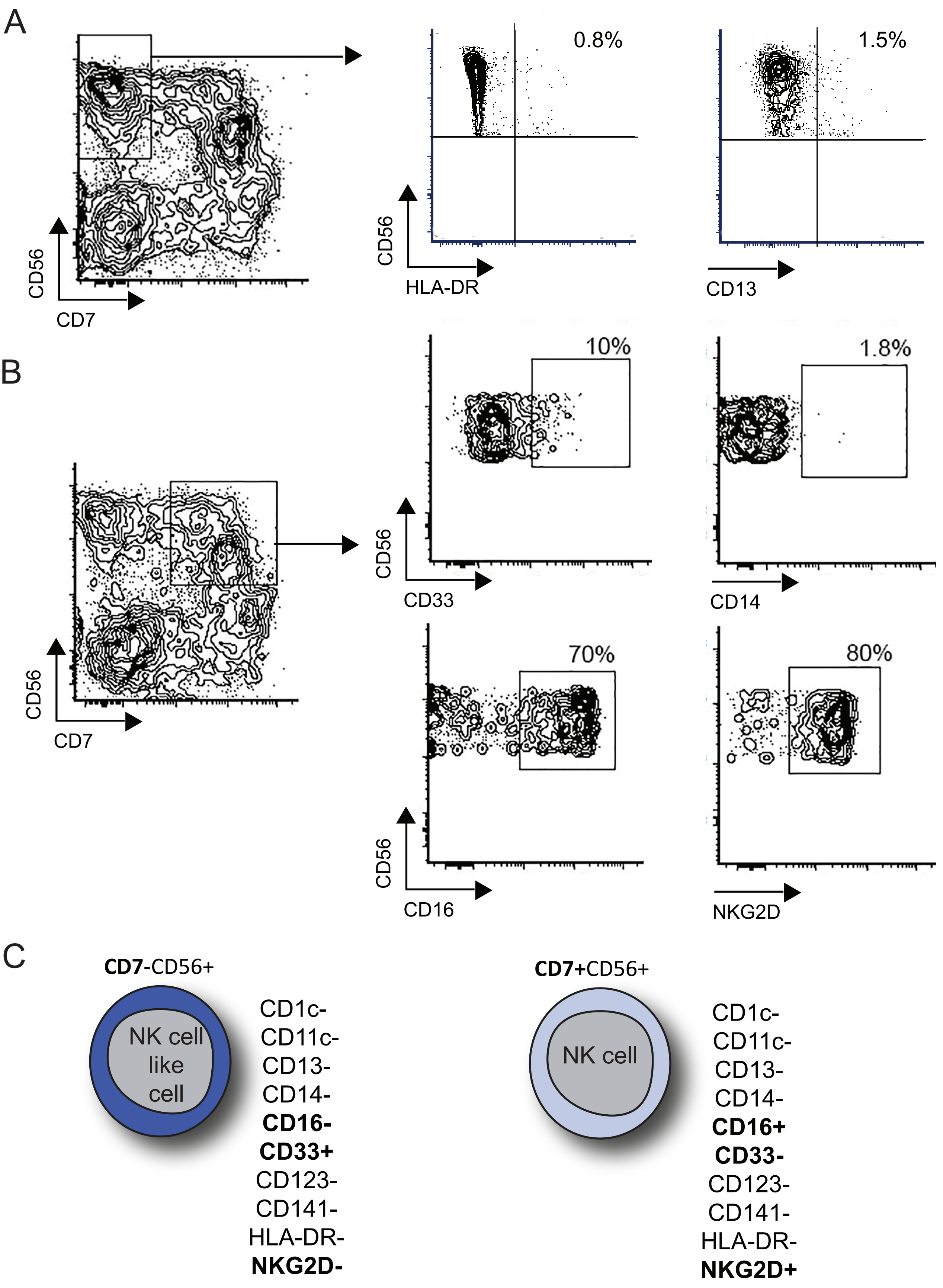

Figure S5. Characterization of *RAG2SCID* iPSC-derived CD7-CD56+ and CD7+CD56+.
A. Expression of HLA-DR and CD13 in CD7-CD56+ cells and B. Expression of myeloid (CD14 and CD33) and NK cell markers (CD16 and NKG2D) in CD7+CD56+ cells. C. Schematic overview of the tested marker profile of *RAG2SCID* iPSC-derived CD7-CD56+ and CD7+CD56+ cells. Differences are indicated in bold.

Table S1. Oligo sequences.

**Primer name**	**Purpose**	**Sequence 5' to 3'**	
coKLF4SOX2for	Provirus detection	GAAGCGACGAGCTGACCCGG	
coKLF4SOX2rev	Provirus detection	TGCAGGGCTCTCAGCCGCTT	
REChRAGMCSupper	RAG recombineering site in pSuperCOS	AAGAATTCGAA**GGCCGGTGGGGACAGGGCTGAGCCAGCACCAACCACTCAGCCTTTGAGA**TATCTA **GACTAACACACTGCCAAAATGTGGCAT**GAATTCAAA	11: 36590857-36590905<br><br>11:36619551-36619577
REChRAGMCSlower	RAG recombineering site in pSuperCOS	TTTGAATTC**ATGCCACATTTTGGCAGTGTGTTAGTCC**TAGATA **TCTCAAAGGCTGAGTGGTTGGTGCTGGCTCAGCCCTGTCCCCACCGGCC**TTCGAATTCTT	
SbfILoxPfor	Creating SbfI sites 5' and 3' of LoxpPGKPurΔTKLoxP	CCTGCAGGACCCTTAATATAACTTCGTATAATGTATG	
SbfILoxPrev	Creating SbfI sites 5' and 3' of LoxpPGKPurΔTKLoxP	CCTGCAGGACCTAATAACTTCGTATAGCATAC	
hRAG2For	HDR analysis	GCAAGACTGTGCAATTCACAGCTGG	11: 36614962-36614986
hRAG2Rev	HDR analysis	CCCTCTGGCCTTCAGGTAGGTCTG	11: 36619643-36619666
hRAG1For_nested	gDNA control	GCAAAGAGGTTCCGCTATGATTCAGC	11: 36596523-36596548
hRAG1Rev	gDNA control	GGCTTGCAACACAGTTCAGAGTTAGG	11: 36596799-36596824
PGKrev	PuΔTK excision analysis	CTTGGCTGGACGTAAACTCCTCTTC	
hRAGPuTKexcFor	PuΔTK excision analysis	CCATCCCACAGCTCCACTAGGCA	11: 36602877-36602899
hRAGPuTKexcRev	PuΔTK excision analysis	CTAACTTGCACAGCCTTTGGAC	11: 36603216-36603237
Vδ1	TCD rearrangements	ATGCAAAAAGTGGTCGCTATT	
Vδ2	TCD rearrangements	ATACCGAGAAAAGGACATCTATG	
Dδ2-5'	TCD rearrangements	AGCGGGTGGTGATGGCAAAGT	
Dδ3-3' (HEX)	TCD rearrangements	TATAGGAGTGGGACCCAGGGT	
