# Supplementary material for: iPSC-Based Modeling of RAG2 Severe Combined Immunodeficiency Reveals Multiple T Cell Developmental Arrests
Source: Stem Cell Reports. 2020 Jan 16;14(2):300–11. doi: 10.1016/j.stemcr.2019.12.010 (PMC7013232; doi:10.1016/j.stemcr.2019.12.010)
Supplement: Document S2. Article plus Supplemental Information [file mmc2.pdf]

Stem Cell Reports
Article
OPEN ACCESS

iPSC-Based Modeling of *RAG2* Severe Combined Immunodeficiency Reveals Multiple T Cell Developmental Arrests

Maria Themeli,[1] Amiet Chhatta,[2] Hester Boersma,[3] Henk Jan Prins,[1] Martijn Cordes,[2] Edwin de Wilt,[4] Aïda Shahrabi Farahani,[1] Bart Vandekerckhove,[5] Mirjam van der Burg,[6,8] Rob C. Hoeben,[3] Frank J.T. Staal,[2] and Harald M.M. Mikkers[3,7,*]
[1]Department of Hematology, Amsterdam UMC, Location VUmc, Cancer Center Amsterdam, Amsterdam 1081 HV, The Netherlands
[2]Department of Immunohematology & Blood Transfusion, Leiden University Medical Center, Leiden 2333 ZA, The Netherlands
[3]Department of Cell & Chemical Biology, Leiden University Medical Center, Leiden 2300 RC, The Netherlands
[4]Department of Clinical Genetics, Leiden University Medical Center, Leiden 2333 ZC, The Netherlands
[5]Department of Clinical Chemistry, Microbiology and Immunology, Ghent University, Gent 9000, Belgium
[6]Department of Immunology, Erasmus Medical Center, Rotterdam 3015 GE, The Netherlands
[7]LUMC hiPSC Hotel, Leiden University Medical Center, Leiden 2333 ZC, The Netherlands
[8]Present address: Department of Pediatrics, Leiden University Medical Center, Leiden 2333 ZA, The Netherlands
*Correspondence: h.mikkers@lumc.nl
https://doi.org/10.1016/j.stemcr.2019.12.010

SUMMARY

RAG2 severe combined immune deficiency (RAG2-SCID) is a lethal disorder caused by the absence of functional T and B cells due to a differentiation block. Here, we generated induced pluripotent stem cells (iPSCs) from a RAG2-SCID patient to study the nature of the T cell developmental blockade. We observed a strongly reduced capacity to differentiate at every investigated stage of T cell development, from early $CD7^-CD5^-$ to $CD4^+CD8^+$. The impaired differentiation was accompanied by an increase in $CD7^-CD56^+CD33^+$ natural killer (NK) cell-like cells. T cell receptor D rearrangements were completely absent in *RAG2SCID* cells, whereas the rare T cell receptor B rearrangements were likely the result of illegitimate rearrangements. Repair of *RAG2* restored the capacity to induce T cell receptor rearrangements, normalized T cell development, and corrected the NK cell-like phenotype. In conclusion, we succeeded in generating an iPSC-based RAG2-SCID model, which enabled the identification of previously unrecognized disorder-related T cell developmental roadblocks.

INTRODUCTION

Severe combined immune deficiency (SCID) is a life-threatening disorder caused by a defective acquired immune system due to the absence of functional T cells (Fischer, 2000). In addition to T cells, SCID patients sometimes lack B cells and/or natural killer (NK) cells depending on the underlying genetic mutations. Milder forms of SCID can also occur as a consequence of hypomorphic mutations. One subgroup of SCID patients, accounting for approximately 30% of the SCID cases, lacks both T and B cells (Gaspar et al., 2013). Mutations causing $T^{neg}B^{neg}$ SCID most often affect genes that play a role in the rearrangement of the B cell receptor (*BCR*) and T cell receptor (*TCR*) loci, such as recombination activating genes (*RAG*) *RAG1* and *RAG2*, *DCLRE1C*, and *PRKDC* (Dvorak and Cowan, 2010). RAG1 and RAG2 proteins form a tetrameric complex of two RAG1/RAG2 heterodimers, of which one is bound to a 12-recombination signal sequence (RSS) and one to a 23-RSS in the V-D-J regions of the *BCR* and *TCR* loci. The tetrameric RAG complex catalyzes the pairwise cleavage of the RSSs, which are connected through non-homologous end joining, creating the huge V-D-J diversity that underlies the enormously diverse immune repertoire (Notarangelo et al., 2016).

Most of our knowledge of the defects in T cell development in SCID has been gathered from gene-knockout studies in mice. However, the differentiation of human T cells follows a slightly different route compared with that of murine T cells (Blom et al., 1999; Dik et al., 2005; Hao et al., 2008; Weerkamp et al., 2006). To fully understand human SCID, and to study therapeutic interventions, an accessible model that faithfully reflects human SCID is required. Modeling human SCID can be performed *in vitro* by culturing primary $CD34^+$ SCID hematopoietic stem/progenitor cells (HS/PCs) on a layer of OP9 cells that express the Notch ligands delta-like 1 (DLL1) or delta-like 4 (DLL4) (Six et al., 2011) or *in vivo* by transplantation of primary long-term repopulating $CD34^+$ SCID hematopoietic stem cells (HSCs) into immune-deficient NOD-SCID common $\gamma^{-/-}$ (NSG) mice (Wiekmeijer et al., 2016). Wiekmeijer and colleagues transplanted HSCs from SCID-X1, IL7R-SCID, and DCLRE1C-SCID patients into NSG animals and observed an earlier block in T cell development than anticipated on the basis of gene expression profiles during human T cell development and corresponding mouse knockouts. This study highlighted that human SCID models are required to investigate the precise underlying developmental defect. However, these experiments fully relied on the availability of primary SCID HS/

 © 2019 The Authors.
This is an open access article under the CC BY license (http://creativecommons.org/licenses/by/4.0/).

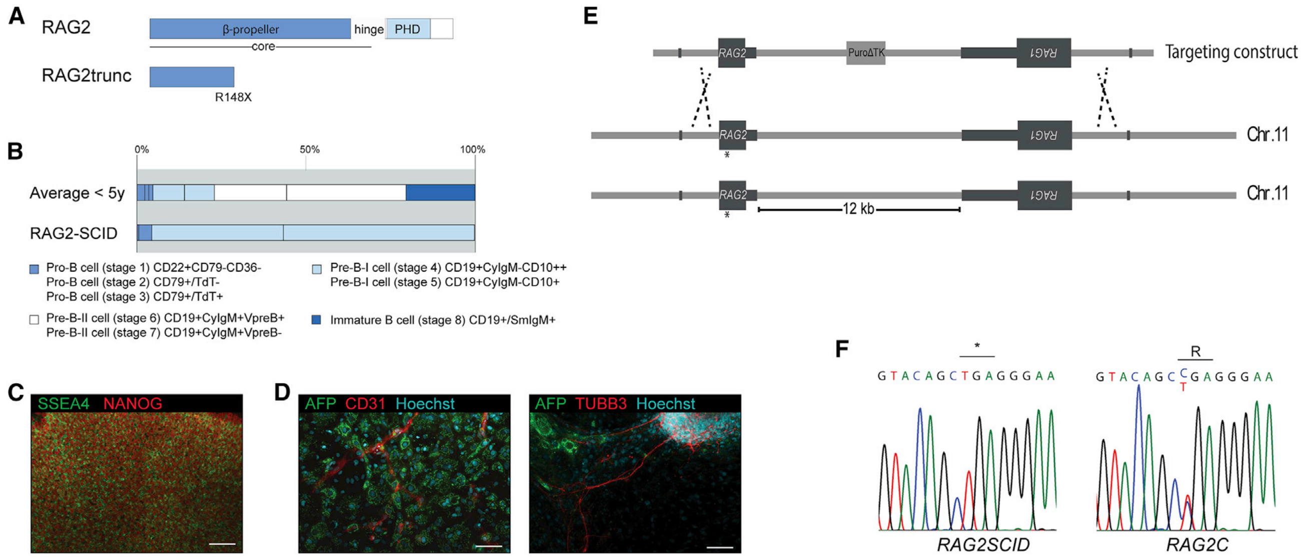

Figure 1. Generation of *RAG2SCID* and *RAG2* Repaired iPSCs

(A) Illustration of the mutant RAG2 p.R148X that causes RAG2-SCID. PHD, plant homology domain.

(B) Percentages of different B cell populations found in the bone marrow of healthy donors of <5 years (upper bar) and of the RAG2-SCID patient with the homozygous mutation depicted in (A) (lower bar).

(C) Fluorescence microscopy analysis of the pluripotency markers NANOG and SSEA4 in one of the generated *RAG2SCID* iPSC clones.

(D) Analysis of the capacity of the generated *RAG2SCID* iPSCs to spontaneously differentiate into derivatives of the three germ layers (AFP, endoderm; TUBB3, ectoderm; CD31, mesoderm) by fluorescence microscopy.

(E) Schematic illustration of the strategy to repair the *RAG2* mutation using homology-directed repair.

(F) *RAG2* sequence analysis of *RAG2SCID* and a repaired *RAG2C* iPSC clone (*RAG2C1*).

Scale bars represent 100 μm.

PCs, which is very restricted due to the rarity of the disorder as well as the wide range of mutations leading to different phenotypes.

Pluripotent stem cells (PSCs) provide a good alternative to model SCID, as human PSCs can be differentiated into T cells *in vitro* (Themeli et al., 2013; Timmermans et al., 2009) and *in vivo* (Galic et al., 2009). Artificial human PSCs can be generated from somatic cells by the overexpression of factors that reset the epigenetic program of somatic cells into that of PSCs (Takahashi et al., 2007; Yu et al., 2007). These so-called induced PSCs (iPSCs) have been successfully used to model SCID-X1 (Menon et al., 2015), JAK3-SCID (Chang et al., 2015), Wiskott-Aldrich syndrome (Laskowski et al., 2016), and RAG1-SCID (Brauer et al., 2016). Since the genetic defect that causes SCID determines the exact stage at which T cell development is stagnated , other SCID disorders should be investigated as well. In addition, the consequences of such a block for the differentiation into other cell types has not been addressed in great detail. Thus it is essential to determine to what extent human iPSC-based SCID models mimic other available SCID models by covering a wide variety of SCID mutations and corresponding phenotypes.

We generated iPSCs from a SCID patient with a homozygous *RAG2* null mutation and demonstrate that *RAG2*-deficient iPSCs have defects throughout every step of T cell development, starting from one of the earliest T-lymphoid-committed stages. The most prominent partial arrests are located at the transition from $CD7^{+}CD5^{-}$ cells into $CD7^{+}CD5^{+}$ T cells and subsequent differentiation steps. Inhibited T cell differentiation is accompanied by a fate shift toward NK cell-like $CD7^{-}CD56^{+}CD33^{+}$ cells. Repair of the mutant *RAG2* gene by homologous recombination restored the differentiation phenotype as illustrated by a normal number of $CD4^{+}CD8^{+}$ double-positive (DP) T cells with polyclonal TCRδ (TCD) and TCRβ (TCB) rearrangements.

RESULTS

Generation of RAG2-SCID iPSCs and Isogenic Control iPSCs

We generated iPSCs from a female RAG2-SCID patient with a homozygous nonsense mutation (p.R148X) in RAG2 (Figure 1A) by transduction of the patient's dermal fibroblasts with a lentiviral vector expressing codon-optimized *OCT4* and *KLF4*, *SOX2*, and *MYC* (Warlich et al., 2011). The selected RAG2-SCID patient demonstrated a complete SCID phenotype indicated by the virtual absence of

B and T cells in the peripheral blood (leukocyte count <0.01 × 10^9/L) and a block in precursor B cell differentiation before the pre-B-II cell stage (Figure 1B). The NK cell count of 0.21 × 10^9/L was normal, indicative of a $T^{neg}B^{neg}NK^{+}$ SCID. The generated iPSC clones expressed the pluripotency markers NANOG, OCT3/4, SSEA4, and TRA1-81 (Figures 1C and S1A) and could spontaneously differentiate into the three germ layers (Figures 1D and S1B). We did not identify very large differences in the hemogenic differentiation potential of the different clones upon coculturing with OP9 cells. The percentage of $CD31^{+}CD34^{+}$ DP cells ranged from 0.4% to 1.7%, which was lower than with control H1 embryonic stem cells (ESCs) (3.9%) but similar to skin-derived iPSCs from a healthy donor (1.5%). This is in line with the observation that genetic background differences are the major contributors to variations in the differentiation potential of iPSC lines (Kajiwara et al., 2012; Kilpinen et al., 2017). We removed the inserted, single-copy, provirus from one of the RAG2SCID clones through hc.fiber50.FLPe adenoviral vector-mediated FLPe expression to avoid a potential position effect of the integrated provirus (Figures S1D and S1E). Karyotype analysis of the generated iPSC clones did not reveal any gross genomic aberrations (Figure S1I). In addition, the pluripotent potential of the *RAG2SCID* iPSC clones was demonstrated by differentiation into ectodermal, endodermal, and mesodermal derivatives *in vivo* (Figure S1C). Subsequently, the *RAG2* mutation was repaired through conventional homologous recombination (Figures 1E, 1F, S1F, and S1G). To ensure that *RAG1* and *RAG2* transcription fully mirrored *RAG* expression in unmodified, wild-type cells, the intergenic PGKpuromycinΔTK selection cassette was removed from one of the repaired iPSC clones by fiber5.CRE adenoviral vector-mediated CRE recombination (Figure S1H). The pluripotent nature and genomic integrity of the repaired *RAG2* iPSC clones (*RAG2C*) were reconfirmed before further assessment of the phenotype (Figures S1C and S1I).

Early Hematopoietic Differentiation Is Unaffected in RAG2-SCID iPSCs

First we wished to compare the capacity of the *RAG2* mutant (*RAG2SCID*) and the *RAG2* repaired (*RAG2C)* iPSC lines to differentiate into early hemogenic endothelial cells and committed hematopoietic progenitors. Early hemogenic colonies from *RAG2SCID* and *RAG2C* were comparable in morphology (Figure S2A). Analysis of the endothelial marker CD31 (Lertkiatmongkol et al., 2016), the hematopoietic and endothelial progenitor marker CD34 (Ditadi and Sturgeon, 2016), the early hematopoietic commitment marker CD43 (Vodyanik et al., 2005), and the universal hematopoietic marker CD45 at day 9 and day 12 of hematopoietic differentiation showed similar hemogenic and committed hematopoietic populations in *RAG2SCID*, *RAG2C*, and control H1 ESCs (Figures 2A, 2B, and S2B).

RAG2-SCID Cells Are Impaired in T Cell Development

Since the RAG2 defect likely affects T cell development, we investigated the differentiation of *RAG2SCID* iPSCs into T cells using the OP9-DLL1 coculture system (Themeli et al., 2013). In *Rag2* knockout mice, the T cell differentiation block is located in $CD4^{-}CD8^{-}CD44^{-}CD25^{+}$ (DN3) cells, yielding dramatically reduced numbers of $CD4^{+}CD8^{+}$ DP T cells (Shinkai et al., 1992). In the three *RAG2SCID* iPSC clones, $CD4^{+}CD8^{+}$ DP T cells were sparsely observed, in contrast to the repaired *RAG2C* iPSC clones and the gold standard H1 ESCs (Figures 3A, 3B, and S3). Immature human T cells are first $CD7^{+}CD5^{-}$ and subsequently become $CD7^{+}CD5^{+}$ prior to differentiation into immature single-positive (ISP) cells. The $CD7^{+}CD56^{-}$ lymphoid population, consisting of $CD7^{+}CD5^{-}$ as well as $CD7^{+}CD5^{+}$ cells, was significantly reduced in *RAG2SCID* cells, indicating that the *RAG2* mutant cells generated $CD7^{+}$ lymphoid cells less efficiently in comparison with H1 and *RAG2C* cells (Figures 3A, 3C, and S3). Dissection of the $CD7^{+}$ lymphoid population showed that the *RAG2SCID* cells are impaired in becoming $CD7^{+}CD5^{+}$ cells and in differentiating further into $CD4^{+}CD8^{+}$ DP T cells (Figures 3A, 3D, 3E, and S3). Thus, it appears that *RAG2SCID* cells are hindered in their progression through different stages of T cell development, with the strongest arrest located at the $CD7^{+}CD5^{+}$ stage.

RAG2SCID T and NK Cells Do Not Undergo Legitimate TCR Rearrangements

Since RAG2 is, together with RAG1, responsible for the generation of double-strand breaks (DSBs) at the border of an RSS required for the rearrangement of the different TCR segments, we investigated the presence and features of TCB rearrangements by GeneScan analysis (Bruggemann et al., 2004). Whereas H1- and *RAG2C*-derived cells undergo normal TCB rearrangement as shown by the presence of polyclonal T cell populations represented by the Gaussian distribution of Vβ-Jβ2(2.2, 2.6, 2.7) fragment sizes, we could not detect any Vβ-Jβ2 rearrangements in *RAG2SCID* $CD7^{+}$ $CD56^{-}$ cells (Figures 4A and S4A). Nevertheless, the presence of a small fraction of $CD4^{+}CD8^{+}$ DP *RAG2SCID* cells in the iPSC-derived T cell differentiation cultures indicates that a few *RAG2SCID* cells could undergo successful positive selection, which is instigated by TCB rearrangements. Therefore, we also investigated the presence of Vβ-Jβ1 rearrangements. We observed very few Vβ-Jβ1 rearrangements in *RAG2SCID* cells (Figure 4B), of which a proportion was of an unexpected size (predicted sizes 240–285 bp). This indicates that the

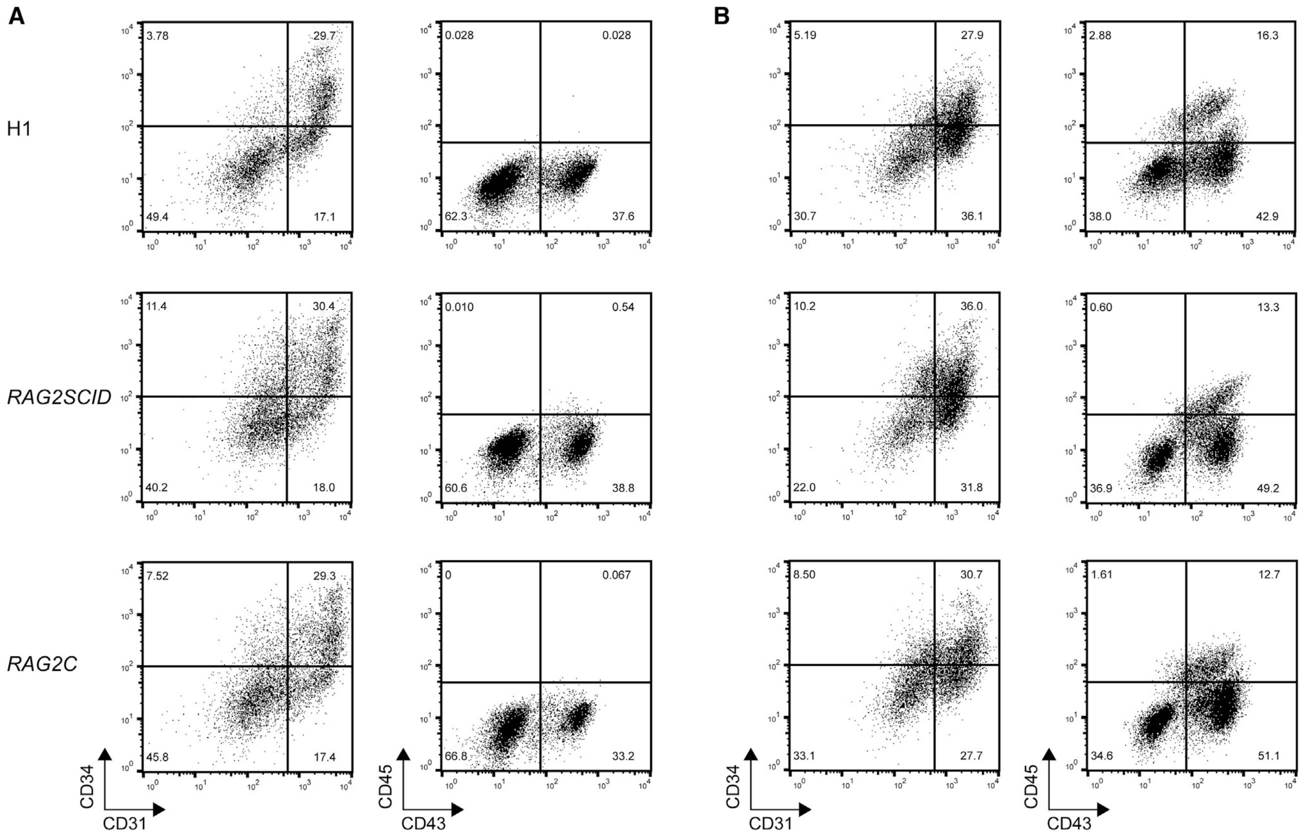

Figure 2. Similar Capacities of *RAG2SCID* and *RAG2C* iPSCs to Differentiate toward Early Hematopoietic Cells *In Vitro*

Flow cytometric analysis of hemogenic and hematopoietic cell types (A) at day 9 and (B) at day 12 of *in vitro* hematopoietic differentiation of H1 (top), *RAG2SCID* (middle), and *RAG2C* cells (bottom). One plot is a representative of the results of three independent experiments.

identified rearrangements are rather the result of the repair of rare spontaneous DSBs than of genuine RAG-driven, RRS-based recombination. To further prove the absence of legitimate rearrangements we checked for the presence of immature TCD rearrangements in developing *RAG2SCID* and *RAG2C* T cells. The first rearrangement during T cell development, also for αβ T cells, is the Dδ2-Dδ3 rearrangement in the TCD locus. Dδ2-Dδ3 rearrangements are already observed in very early $CD34^{+}CD38^{-}$ thymocytes and peak in $CD34^{+}CD38^{+}$ thymocytes (Dik et al., 2005). Dδ2-Dδ3 rearrangements are subsequently followed by immature Vδ2-(Dδ1-Dδ2)-Dδ3 rearrangements. We observed Dδ2-Dδ3 and Vδ2-Dδ3, as well as more mature Vδ1-Dδ3, TCD rearrangements in sorted $CD7^{+}CD56^{-}CD4^{-}CD8^{-}$ *RAG2C* cells but not in sorted $CD7^{+}CD56^{-}$ *RAG2SCID* T cells or in *RAG2C* iPSCs (Figures 4C and S4B). Comparison of the TCD rearrangements in unsorted populations of differentiated *RAG2C* cells and H1-derived cells showed very similar Dδ2-Dδ3, Vδ2-Dδ3, and Vδ1-Dδ3 profiles, in terms of both clonality and size distributions (Figure 4D). Of note, Dδ2-Dδ3 rearrangements are more diverse in primary human ISP cells (Figure S4B). Cells that are similar to T cells and are generated in, among others, the thymus are NK cells (Narni-Mancinelli et al., 2011). In particular, $CD56^{bright}$ cells represent an intrathymic NK cell population that is responsive to interleukin-7 (IL-7) (Michaud et al., 2010; Vosshenrich et al., 2006). One NK cell subpopulation, which is thought to have higher fitness than the others, at least in mice, consists of NK cells that express RAG during a certain stage of their development (Karo et al., 2014). As a consequence, a proportion of NK cells, also in men, exhibits TCD rearrangements, in particular immature Vδ2-Dδ3 rearrangements (Fronkova et al., 2005). Since the OP9-DLL1 differentiation protocol used supports the development of $CD7^{+}CD56^{+}$ NK cells (Zeng et al., 2017) (Figure 3A), we checked TCD rearrangements in $CD7^{+}CD56^{+}$ cells derived from *RAG2SCID* and *RAG2C* iPSCs. Vδ2-Dδ3 rearrangements were prominent in *RAG2C* $CD7^{+}CD56^{+}$ cells, confirming the results found in primary human NK cells (Figure 4E). We could also detect Vδ1-Dδ3 rearrangements in *RAG2C* $CD7^{+}CD56^{+}$ cells, whereas the most immature Dδ2-Dδ3 rearrangements were largely absent (Figure 4E). In contrast,

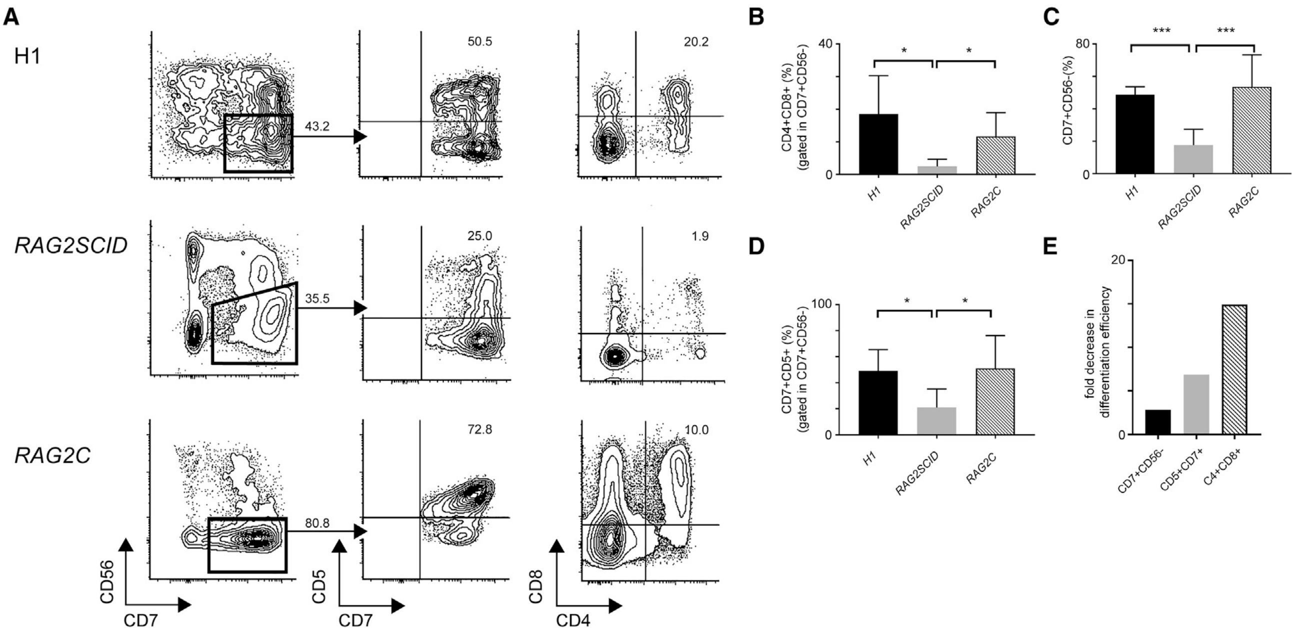

Figure 3. Impaired Differentiation of *RAG2SCID* iPSCs into T Cells *In Vitro*

(A) Flow cytometric analysis of the differentiation of H1 (top), *RAG2SCID* (middle), and *RAG2C* (bottom) cells into the T lineage.

(B–D) (B) Plotted percentages of generated $CD4^+CD8^+$ DP T cells, (C) early $CD7^+$ lymphoid cells, and (D) $CD7^+CD5^+$ cells.

(E) Fold decrease in the differentiation of *RAG2SCID* iPSCs into T lineage cells compared with the repaired *RAG2C* iPSCs. Results in (B), (C), (D), and (E) are from three independent experiments using 3 *RAG2SCID* and 2 *RAG2C* clones. Averages + standard deviations are shown. $*p < 0.05$, $***p < 0.001$

RAG2SCID $CD7^+CD56^+$ cells did not have any TCD rearrangements (Figure 4E). These results highlight that *RAG2SCID* cells are indeed unable to properly recombine the TCR loci in contrast to repaired *RAG2C* cells.

Differentiation of RAG2-SCID Cells Is Skewed toward NK Cell-like Cells

Next, we wished to investigate whether the reduction in $CD7^+$ lymphoid progenitors in *RAG2SCID* cultures was caused by a fate switch of early lymphoid progenitor cells toward cell types that do not require functional TCR rearrangements for their development. Since the OP9-DLL1 differentiation protocol used supports efficient $CD56^+$ NK cell development, we zoomed in on the $CD56^+$ populations. We did not observe alterations in the percentages of $CD7^+CD56^+$ cells generated from *RAG2SCID*, *RAG2C*, or H1 ESCs (Figure 5A). Instead, we found a significant increase in the generation of *RAG2SCID* $CD7^-CD56^+$ cells (Figure 5B). To our knowledge the only described $CD7^-CD56^+$ cells are monocytic/dendritic cell (DC)-like cells that are positive for CD13, CD33, CD123, and HLA-DR (Milush et al., 2009). We analyzed the $CD7^-CD56^+$ population for the expression of a variety of DC, myeloid cell, and NK cell markers by flow cytometry. The iPSC-derived $CD7^-CD56^+$ population was largely positive for CD33, whereas any other tested monocytic cell, DC, and NK cell markers were absent (Figures 5C, S5A, and S5C). In contrast, the $CD7^+CD56^+$ cells showed a typical NK cell profile, as they expressed CD16, which is commonly found on NK cells, and the NK cell marker NKG2D (Figures S5B and S5C). Since myeloid cell and DC markers were absent, we wondered whether the $CD7^-CD56^+CD33^+$ cells would respond to NK cell-specific stimuli. $CD7^-CD56^+$ cells stimulated with IL-12, IL-15, and IL-18 produced similar levels of IFN-γ, TNF-α, and GM-CSF compared with iPSC-derived typical $CD7^+CD56^+$ NK cells (Figure 5D). Only the "mutually exclusive" production of IL-10 and IL-13 allowed discrimination of the $CD7^-CD56^+$ and $CD7^+CD56^+$ populations (Figure 5D). In conclusion, our results suggest that inhibition of T cell development by RAG2 deficiency leads to a skewed differentiation toward $CD7^-CD56^+CD33^+$ NK cell-like cells.

DISCUSSION

SCID is caused by a wide variety of mutations and has been mainly investigated using mouse knockout models. It has become evident that human SCID models using mouse knockouts of the affected gene do not represent the human

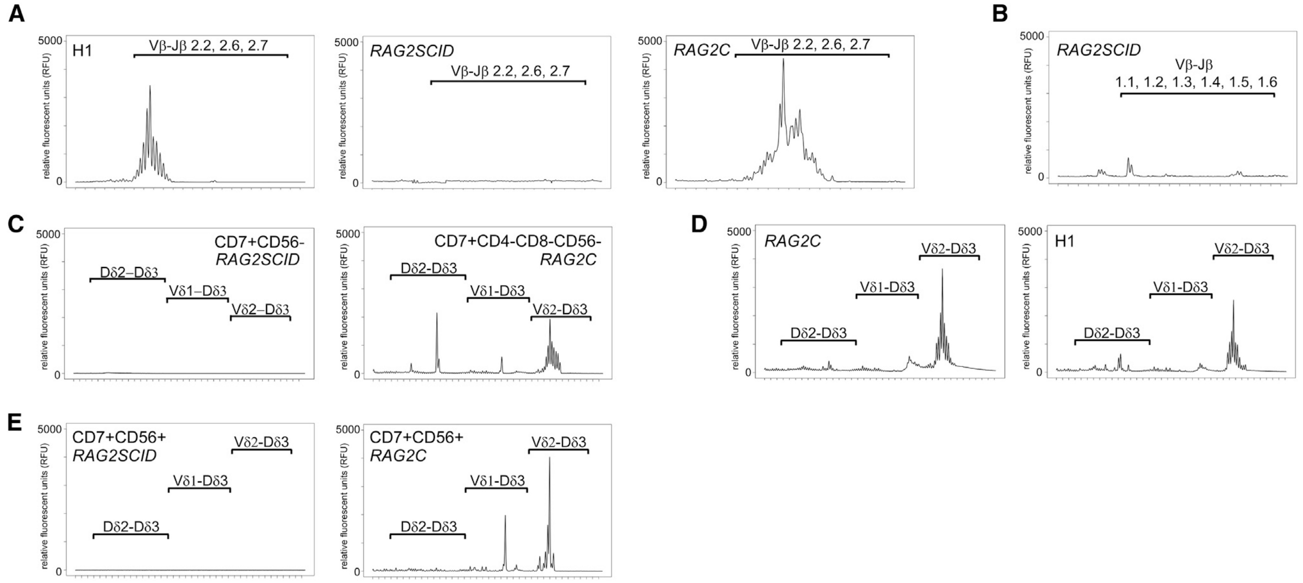

Figure 4. Defective T Cell Receptor Rearrangements in *RAG2SCID* Cells Are Rescued by Repair of *RAG2*

(A) GeneScan analysis of TCB (Vβ-Jβ2) rearrangements in whole populations (H1 and *RAG2C*) and in sorted $CD7^{+}CD56^{-}$ cells (*RAG2SCID*).

(B) GeneScan analysis of TCB (Vβ-Jβ1) rearrangements in sorted $CD7^{+}CD56^{-}$ cells (*RAG2SCID*).

(C) GeneScan analysis of TCD rearrangements in sorted $CD7^{+}CD56^{-}$ cells (*RAG2SCID*) and $CD7^{+}CD4^{-}CD8^{-}CD56^{-}$ cells (*RAG2C*).

(D) GeneScan analysis of TCD rearrangements in whole populations (H1 and *RAG2C*).

(E) GeneScan analysis of TCD rearrangements in $CD7^{+}CD56^{+}$ cells of *RAG2SCID* and *RAG2C*. Fragment length (TCD 150–268 bp and TCB 220–289 bp) is plotted on the x axis. All samples analyzed were from week 4–5 differentiation cultures.

situation very well, likely because human T cell development is different from that in mice (Blom et al., 1999; Dik et al., 2005; Hao et al., 2008; Weerkamp et al., 2006). The best human SCID model to date involves the transplantation of $CD34^{+}$ SCID HS/PCs into NSG animals (Wiekmeijer et al., 2016). However, the availability of $CD34^{+}$ HS/PCs from SCID patients is very limited due to the rarity of the disorder and the wide range of different SCID mutations. Therefore an alternative SCID modeling system is warranted. iPSCs derived from SCID patients have shown promise, but due to the wide variety of SCID mutations and corresponding phenotypes, additional studies are required to evaluate iPSC-based SCID modeling. Using RAG2-SCID iPSCs we observed phenotypic differences with the reported phenotype of *Rag2* knockout mice (Shinkai et al., 1992). *RAG2SCID* iPSC-derived cells showed a partial arrest at every stage of T cell development, ultimately leading to very few $CD4^{+}CD8^{+}$ DP T cells (Figure 6). The transitions from $CD7^{+}CD5^{-}$ into $CD7^{+}CD5^{+}$ cells and from $CD7^{+}CD5^{+}$ into $CD4^{+}CD8^{+}$ T cells were most profoundly impaired in *RAG2SCID*. Our results are in line with the results obtained from the transplantation of primary bone marrow DCLRE1C-SCID $CD34^{+}$ hematopoietic cells into NSG mice, where a full block at the $CD7^{+}CD5^{+}$ stage and a reduced differentiation into $CD7^{+}CD5^{-}$ as well as $CD7^{+}CD5^{+}$ cells were observed (Wiekmeijer et al., 2016). Artemis, which is encoded by *DCLRE1C*, is involved in opening of the hairpins formed at both nicked RSSs, which are required for V(D)J recombination (Ma et al., 2002). As a consequence the pathogenic phenotypes of RAG-SCID and DCLRE1C-SCID are very similar (Dvorak and Cowan, 2010). Despite the lack of functional Artemis the absence of $CD4^{+}CD8^{+}$ DP T cells, TCB and TCD rearrangements were found in the arrested-DCLRE1C-SCID cells, possibly because the hairpins were resolved by a repair mechanism other than Artemis (Wiekmeijer et al., 2016). In contrast, *RAG2SCID* iPSC-derived cells showed only very rare TCB rearrangements, yet immature Dδ2-Dδ3 rearrangements were undetectable. From our data it could be argued that immature TCD rearrangements, which are already detected in the earliest $CD34^{+}CD38^{-}$ T progenitor cells (Dik et al., 2005), contribute to a more efficient initiation and progression of T cell development. A genome-wide association study has indeed revealed that a single-nucleotide polymorphism (SNP) within the Dδ2-Dδ3 region influences T cell numbers, particularly of early $CD4^{-}CD8^{-}$ thymocytes, highlighting the importance of the region between the Dδ2 and the Dδ3 segments (Clave et al., 2018). Since we did not observe a full block at the $CD7^{+}CD5^{+}$ stage, this would indicate that human SCID models based on the coculture of SCID iPSCs and OP9-DLL cells are more lenient. A recent study using

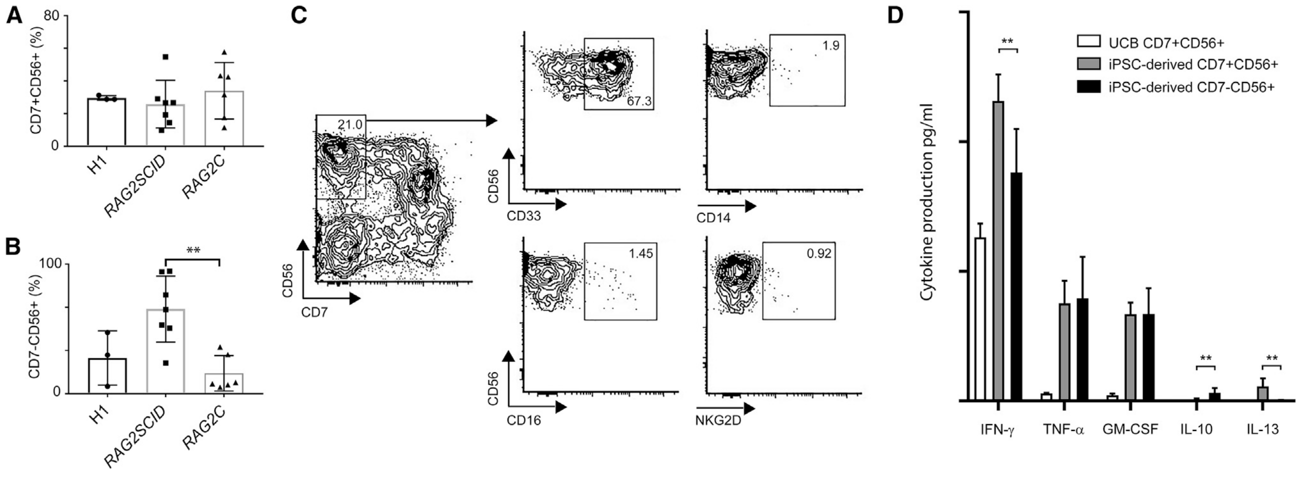

Figure 5. An Increase in CD7$^-$CD56$^+$CD33$^+$ NK Cell-like Cells in *RAG2SCID* iPSC Differentiation Cultures
(A and B) (A) Plotted percentages of iPSC-derived CD7$^+$CD56$^+$ NK cells and (B) CD7$^-$CD56$^+$ cells.
(C) Flow cytometric analysis of CD33 and CD14 (myeloid) and CD16 and NKG2D (NK cell) expression in the CD7$^-$CD56$^+$ population.
(D) Production of cytokines by CD7$^-$CD56$^+$ and CD7$^+$CD56$^+$ cells measured after 20 h of stimulation with IL-12, IL-15, and IL-18. Results are from at least three independent experiments. Averages + standard deviations are shown. **p < 0.01.

RAG1-SCID iPSCs has suggested that multiple T cell waves exist *in vitro* and that only a longer T cell differentiation period of 4–5 weeks better reflects the *in vivo* phenotype (Brauer et al., 2016). In our study we observed very few ISP and DP cells in RAG2-SCID cultures, irrespective of differentiation length. Possibly this difference is explained by the severity of the mutation and/or the affected gene. In addition, the transition from CD3$^-$ DP to CD3$^+$ DP cells may be affected in RAG2-SCID patients, but this remains to be studied. Nevertheless, we describe and rescue similar blocks at the CD7$^+$CD5$^-$ and CD7$^+$CD5$^+$ stages of T cell development, as reported in the RAG1-SCID iPSC-based model.

The absence of TCD rearrangements and the presence of sporadic Vβ-Jβ1 rearrangements, of which a fraction yielded smaller PCR fragment sizes than expected, indicate that the sparse TCB rearrangements found in *RAG2SCID* T lymphoid cells are likely the result of extremely rare random deletions that occurred in only very few cells. These illegitimately rearranged cells are able to bypass the β-selection checkpoint, allowing clonal expansion until the CD4$^+$CD8$^+$ DP stage. At this stage TCRA needs to rearrange, which is likely to fail in *RAG2* mutant cells, resulting in the lack of mature CD4 and CD8 single-positive (SP) cells. In addition to the T cell phenotype, we observed a strong increase of CD7$^-$CD56$^+$CD33$^+$ cells that do not express other common myeloid lineage, DC, and NK cell markers. However, the observed NK cell-specific cytokine production upon stimulation implies an NK cell-like function. NK cells are generated in multiple tissues, among which is the thymus (Yu et al., 2013). In particular CD56^{bright} cells are believed to represent thymic NK cells (Vosshenrich et al., 2006). Recently, a multicenter study showed that CD56^{bright} NK cells are strongly enriched in the periphery of RAG-SCID patients and that these cells have a rather immature phenotype (Dobbs et al., 2017). It would be interesting to see whether the CD56^{bright} NK cells of RAG-SCID patients encompass a CD7$^-$CD56$^+$CD33$^+$ NK cell-like subset as well. Although the human data contrast with the more mature phenotype of NK cells found in *Rag2*-null mice (Karo et al., 2014), it is obvious that the absence of either *RAG* gene has an effect on the NK cell population. The expression of RAG proteins, somewhere along the route of NK cell differentiation, produces TCD rearrangements in a subset of NK cells. In particular, immature Vδ2-Dδ3 rearrangements are frequently found in NK cells in humans (Fronkova et al., 2005). Likewise, we identified predominantly Vδ2-Dδ3 rearrangements in *RAG2*-corrected CD56$^+$CD7$^+$ NK cells, but not in *RAG2SCID* NK cells. This reconfirms the rearrangement deficiency in *RAG2SCID* cells and highlights that the applied iPSC-based model system reflects the *in vivo* situation very well.

In conclusion, we present a human RAG2-SCID model that better recapitulates the mutant *RAG2* phenotype than the equivalent mouse knockout, as we could reveal previously unrecognized blocks in T cell development. Although direct comparison with other *in vivo* human SCID modeling systems is still needed, this model likely provides opportunities to link specific mutations to the wide variety of human SCID phenotypes and to evaluate the ability of drugs to rescue the observed phenotype.

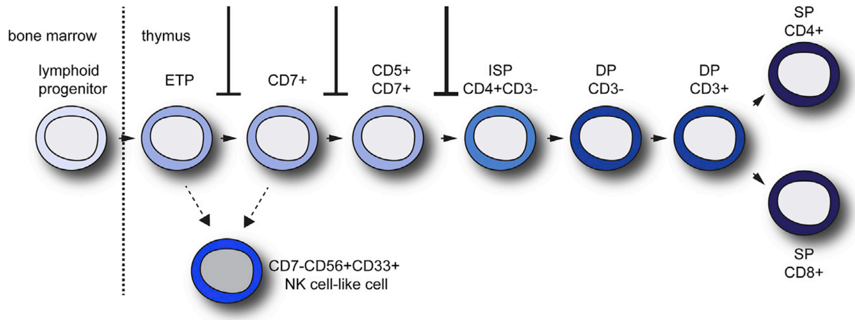

Figure 6. Schematic Illustration of the Impaired T Lineage Differentiation in RAG2-SCID

On the basis of our findings we propose partial developmental arrests at several stages of T cell development. The extent of inhibition is represented by line width. As a consequence of the impaired capacity to progress through T cell development, $CD7^{-}CD56^{+}CD33^{+}$ NK cell-like cells are likely generated from early $CD7^{-}$ or $CD7^{+}$ progenitors.

EXPERIMENTAL PROCEDURES

Generation and Characterization of iPSCs

Passage three fibroblasts from a female RAG2-SCID patient were reprogrammed into iPSCs as described elsewhere (Dambrot et al., 2013). The patient contained a normal number of NK cells in the peripheral blood, whereas B and T cells were <0.01 × 10^9/L. In the bone marrow there was a complete block prior to the cytoplasmic Igμ-positive pre-B-II stage. In brief: 2 × 10^4 human fetal fibroblasts were transduced at an efficiency of <10% with the multicistronic lentiviral vector LV.RRL.PPT.SF.hOKSM.idTomato.-preFRT (Warlich et al., 2011) in a 12-well plate. One day after transduction, fresh human fibroblast medium (DMEM/F12, 10% fetal bovine serum [FBS] [Thermo Fisher Scientific], non-essential amino acids [NEAA] [Thermo Fisher Scientific], 100 μM β-mercaptoethanol [Sigma], and penicillin/streptomycin) was added. Cells were harvested using 1× trypsin/EDTA (Thermo Fisher Scientific) 6 days posttransduction, and 1 × 10^4 cells were seeded onto irradiated CD1 murine embryonic fibroblasts (MEFs) in a 10-cm Petri dish (Greiner) coated with 0.1% gelatin (Sigma) in PSC growth medium (DMEM/F12 [Thermo Fisher Scientific], 20% knockout replacement serum [KRS] [Thermo Fisher Scientific], 10 ng/mL human basic fibroblast growth factor [bFGF] [Peprotech], NEAA [Thermo Fisher Scientific], 100 μM β-mercaptoethanol [Sigma], and penicillin/streptomycin [Thermo Fisher Scientific]). The medium was replaced every other day until the appearance of human ESC-like colonies. iPSC clones were manually picked and further expanded on ESC-qualified Matrigel (Becton Dickinson [BD])-coated 6-well plates in mTESR1 (STEMCELL Technologies). After five passages in mTESR1 the clones were also adapted to feeder conditions and further cultured as described below. For a full description of the characterization protocol we refer to previous studies (Chen et al., 2017; Dambrot et al., 2013). Selected clones were analyzed for the expression of pluripotency markers using immunostaining with primary antibodies recognizing OCT3/4 (1:100; Santa Cruz Biotechnology, cat. no. sc-5279), SSEA4 (1:30; Biolegend, cat. no. 330402), TRA1-81 (1:125; Biolegend, cat. no. 330702), and NANOG (1:500; R&D Systems, cat. no. 963488) and Alexa 488-, Alexa 568-, and Alexa 647-conjugated secondary antibodies (1:500; Thermo Fisher Scientific). Differentiation into three-germ-layer derivatives was assessed by spontaneous differentiation in MEF medium for 3 weeks and subsequent detection of expression of germ-layer-specific markers using immunofluorescence microscopy. Antibodies against AFP (1:25; Quartett, cat. no. 2011200530), CD31 (1:100; Dako, cat. no. M0823), and TUBB3 (1:4,000; Covance, cat. no. MMS-435P) were used. Secondary antibodies were conjugated with either Alexa 488 or Alexa 568 (1:500; Thermo Fisher Scientific). The genome of the clones was analyzed with combined binary ratio labeling (COBRA).

The number of provirus insertions was determined by Southern blot analysis using 10 μg of PaeI-restricted genomic DNA and a NotI/HincII fragment containing the RRE part of the lentiviral reprogramming vector as probe.

To generate teratomas 1 × 10^6 cells were injected subcutaneously into 8- to 12-week-old immunocompromised female *Rag2*$^{-/-}$ mice. Teratomas were isolated 10–16 weeks postinjection. Teratomas were fixed in 4% paraformaldehyde and, subsequently, embedded in paraffin and sectioned. Ectodermal, endodermal, and mesodermal derivatives were visualized by H&E staining and standard light microscopy.

All human materials were collected according to the approval by the Medical Ethics Committee of Erasmus MC (reference no. MEC-2016-606) or LUMC (reference no. P08-087). The experiments involving human materials were done in accordance with the principles outlined in the Declaration of Helsinki. Animal experiments were approved by the Animal Experiments Committee of LUMC (reference no. 12133) and were performed following the recommendations and guidelines set by LUMC and the Dutch Experiments on Animals Act.

Maintenance Culture of PSCs

RAG2SCID iPSC clones (official names: LUMC043iRAG04, LUMC043iRAG05, LUMC043iRAG06, and LUMC043iRAG18), the repaired isogenic controls (LUMC043iRAGC1 and LUMC043iRAGC2), and the wild-type human ESC line H1 (WiCell Research Institute) were cultured on ESC-qualified Matrigel (BD) in mTESR1 (STEMCELL Technologies) or on irradiated MEFs (10,000 cells/cm^2) (either CD1 [maintenance, genetic modification] or CF1 [T cell differentiation]) on 0.1% gelatin (Sigma)-coated tissue culture plastic in human ESC medium (HESCM) consisting of DMEM/F12 (Gibco), NEAA (Thermo Fisher Scientific), 100 μM β-mercaptoethanol (Sigma), 10 ng/mL human bFGF (Peprotech), penicillin/streptomycin, and 20% KRS (Thermo Fisher Scientific). Cells cultured in mTESR1 were refed once a day and passaged once a week using gentle cell dissociation reagent (GCDR) (STEMCELL Technologies). Cells cultured on feeders were refed once every other day and passaged once a week by cut-and-paste or manual dissociation

using either dispase II (Sigma) or collagenase I (Sigma) treatment. For differentiation experiments *RAG2* iPSC lines beyond passage 10 and H1 ESCs beyond passage 27 were used.

Genomic DNA Isolation

Genomic DNA was extracted from iPSC clones or differentiated cells that were snap frozen as follows: cells were incubated in lysis buffer containing 10 mM Tris-HCl (pH 8.0), 25 mM EDTA (pH 8.0), 0.5% (w/v) SDS, 100 mM NaCl, and 100 μg/mL proteinase K (Thermo Fisher Scientific) at 55°C for at least 5 h, after which the DNA was precipitated with 0.7 volumes of isopropanol. For Southern blotting we included one phenol:chloroform:isoamyl alcohol (25:24:1) (Sigma) extraction step prior to precipitation. The precipitated DNA was washed once with 70% (v/v) ethanol and dissolved in 10 mM Tris-HCl (pH 8).

Removal of Provirus

LUMC043iRAG18 was selected for removal of the inserted provirus. To this end the iPSC line was first adapted to single-cell passaging as follows. iPSC colonies were dissociated into single cells or small clumps of cells (1–5 cells) by a 1–3 min incubation with TrypLE select (Thermo Fisher Scientific) and replated in 50% fresh HESCM and 50% old HESCM in which the cells were cultured in the presence of 10 μM Fasudil (LC Laboratories). Irradiated CD1 MEFs were seeded on 0.1% gelatin-coated plates 1 day before passaging. Cells were passaged when they were subconfluent, i.e., iPSC colonies almost touching (either next day or after 2 days). The provirus was removed by transduction of single cells in suspension with hcAd.FLPe.F50 adenoviral vector (Gonçalves et al., 2008) at a multiplicity of infection (MOI) of 20 transduction units (TU)/mL. After 1 h at 37°C, single cells were seeded onto MEFs. Removal of the provirus was determined by PCR analysis of the genomic DNA isolated from single-cell-derived clones 11–12 days after transduction.

Genetic Modification of RAG2SCID iPSCs

RAG2SCID iPSCs were cultured on mTESR1 and Matrigel for the first passages. After passage 5 the iPSCs were cultured on irradiated CD1 MEF feeders (10,000 cells/cm^2) on 0.1% gelatin (Sigma)-coated tissue culture plastic in HESCM. Prior to electroporation, cells were adapted to single-cell passaging for at least three passages as described above. One day prior to electroporation, cells were passaged in the presence of 10 μM Fasudil (LC Laboratories) to reach a subconfluent density the next day. Single cells (1.5×10^7) were electroporated with 40 μg of PvuI (Fermentas)-linearized *RAG* targeting vector in 800 μL of ice-cold PBS in a 0.4 mL cuvette (Bio-Rad) using a Bio-Rad GenePulser II electroporator (250 V, 500 μF). The targeting vector contained a 28,721 bp *RAG1* and *RAG2* genomic DNA fragment from BAC RP11-669J23 (obtained from the Sanger Institute) that was shuttled into pSuperCOS containing *RAG*-recombineering sequences using pRedET (Gene-Bridges)-mediated recombineering. To enable selection, a PCR-amplified SbfI-*LoxP.PGKpuroDelTK.LoxP*-SbfI cassette was inserted into the SbfI site in the intragenic region between *RAG1* and *RAG2*, generating a 5′ homology arm of 12,230 bp and a 3′ homology arm of 16,491 bp. Electroporated cells were seeded onto puromycin-resistant MEFs (transduced with a CMV-puro lentiviral vector) in HESCM containing 10 μM Fasudil (LC Laboratories). Cells were selected in HESCM containing 1 μg/mL puromycin (Thermo Fisher Scientific) for 7 days starting 2 days after electroporation. After 11–12 days colonies were numbered, and half of the colony was collected by scraping with a pipet tip from which genomic DNA was isolated. Correctly recombined clones were identified by PCR amplification of the mutated *RAG2* regions using Phusion polymerase (New England Biolabs), followed by PvuII (Fermentas) restriction of the amplified fragments. The PCR fragments were Sanger sequenced. The clones in which one of the mutant *RAG2* alleles was repaired were expanded and officially named LUMC043iRAGC1 and LUMC043iRAGC2. After repair by homologous recombination, LUMC043iRAGC1 was again adapted to single-cell passaging and the selection cassette was removed using CRE recombinase delivery via a first-generation Ad.CRE.F5 vector at an MOI of 50 TU/mL. Cells from which the selection cassette was removed were selected using 200 nM FIAU (Moravek) for 5 days starting 2 days after viral transduction. Removal of the PuroΔTK marker was verified by PCR using intergenic *RAG* and PGKrev primers and genomic DNA control primers (Table S1).

Hematopoietic Differentiation of iPSCs

The hemogenic potential of the different iPSC clones was determined by coculturing the iPSCs (embryoid bodies [EBs]) on confluent OP9 stroma as described elsewhere (Timmermans et al., 2009). For comparison of the repaired versus parental *RAG2SCID* lines we used the StemDiff hematopoietic kit (STEMCELL Technologies). In brief, we cut iPSC colonies (cultured on hESC-qualified Matrigel [BD] in mTESR1) into small 50–100 μm pieces. These pieces were collected in mTESR1 after short treatment with GCDR (STEMCELL Technologies) and seeded onto Matrigel-coated plates at a density of 4–10 pieces/cm^2. Medium was changed according the manufacturer's instructions and cells were harvested at day 9 and day 12 of hematopoietic differentiation using TrypLE select (Thermo Fisher Scientific). Differentiation was determined by flow cytometry using anti-CD31 BV605 (BD, no. 562855), anti-CD34 PeRCPe710 (eBioscience, no. 46-0349-42), CD43-FiTC (BD, no. 555475), and anti-CD45 V450 (BD, no. 560368). iPSCs incubated with antibodies and unstained differentiated cells were used as negative controls to determine the gates.

iPSCs were differentiated toward the T lymphoid lineage using a previously described protocol (Themeli et al., 2013). Briefly, undifferentiated iPSC colonies were treated with dispase and transferred to low-attachment plates to allow the formation of EBs in Stem-Pro-34 medium (Thermo Fisher Scientific) supplemented with 2 mM L-glutamine, 1% NEAA, 10 μM β-mercaptoethanol, 100 U/mL penicillin and 100 ng/mL streptomycin, and 50 μg/mL ascorbic acid (Thermo Fisher Scientific). The formation of EBs was facilitated by an overnight incubation in the presence of 30 ng/mL hBMP-4 (R&D Systems [R&D]). EBs were then cultured with hBMP-4 (30 ng/mL) (R&D) and hbFGF (5 ng/ml) (Peprotech) until day 4 to allow for mesoderm induction. Next, hematopoietic specification and expansion were achieved in the presence of hVEGF (20 ng/mL) (Peprotech) and a cocktail of hematopoietic cytokines (hSCF, 100 ng/mL [R&D]; hFlt3L, 20 ng/mL; hIL-3, 20 ng/mL; and hIL-6, 10 ng/mL [all Peprotech]). Day 9 EBs containing hematopoietic progenitor cells were dissociated by treatment with Accutase

(Thermo Fisher Scientific) for 20 min, and single cells were then seeded on OP9-DLL1 monolayers to allow for their T lymphoid differentiation in OP9 medium (α-MEM with 20% FBS, 2 mM L-glutamine, 1% NEAA, 10 μM β-mercaptoethanol, 100 U/mL penicillin and 100 ng/mL streptomycin, and 50 μg/mL ascorbic acid [all Thermo Fisher Scientific]) supplemented with SCF, 10 ng/mL (R&D); IL-7, 5 ng/mL (Peprotech); and Flt3L, 10 ng/mL (Peprotech). Once a week cells were harvested and subcultured on OP9-DLL1 monolayers. Medium was replaced once every other day. Differentiation was determined by flow cytometry using the following antibodies: anti-CD1c FiTC, anti-CD4 BV650 (BD, no. 56387), anti-CD5 PE (BD, no. 561897), anti-CD7 PE-Cy7 (eBioscience, no. 25-0079-41), anti-CD8a FiTC (BD, no. 561947), anti-CD11c PerCP-Cy5.5 (BD, no. 565227), anti-CD13 V450 (BD, no. 561157), anti-CD14 APC-Cy7 (BD, no. 557831), anti-CD16 APC-Cy7 (BD, no. 561726), anti-CD33 BV421 (BD, no. 562854), anti-CD43 FiTC (BD, no. 555475), anti-CD45 V450 (BD, no. 560368), anti-CD56 BV785 (Biolegend, no. 362550), CD123 PE (BD, no. 340545), anti-CD141 APC (BD, no. 564123), anti-HLA-DR APC-H7 (BD, no. 561358), anti-NKp46 PE (BD, no. 557991), and anti-NKG2D APC (BD, no. 562064). iPSCs incubated with antibodies and unstained differentiated cells were used as negative controls to determine the gates.

Statistical Analyses

Statistical analyses were performed using GraphPad Prism software version 7.0. For normal distributions two-tailed Student's t tests were used. A p value of <0.05 was considered statistically significant.

TCB and TCD GeneScan Analysis

Genomic DNA was directly isolated from frozen sorted and unsorted cells as described above, except the phenol extraction was omitted. Vβ-Jβ rearrangements were amplified using TCRB tube A (Invivoscribe), Amplitaq GOLD (Thermo Fisher Scientific), and Buffer II (Thermo Fisher Scientific) according to the manufacturer's protocol. The TCD rearrangements were detected using a customized primer mix (Invivoscribe) containing 10 pmol of HEX Dδ3-3′ and Vδ1, Vδ2 Dδ2-5′ primers, Amplitaq GOLD (Thermo Fisher Scientific), 2 mM $MgCl_2$, dNTP (200 μM each), and Amplitaq GOLD Buffer II (Thermo Fisher Scientific). DNA input was 100 ng and PCR conditions were as follows: 7 min at 95°C and 40 cycles 45 s at 95°C, 45 s at 60°C, 35 s at 72°C. Fragments were separated on a 3730 DNA analyzer (Applied Biosystems), analyzed using the R package Fragman (version 1.0.9), and plotted using R version 3.4.4.

NK Cell Stimulation

Cells were isolated from umbilical cord blood or iPSC T cell differentiation cultures by fluorescence-activated cell sorting on the basis of CD7 and CD56 expression. Cells were cultured in AIM V medium (no. 31035025, Thermo Fisher Scientific) containing 5% heat-inactivated FBS without or with 10 ng/mL IL-12 (Peprotech), 10 ng/mL IL-15 (R&D), and 20 ng/mL IL-18 (MBL International) in a 96 round-bottom well plate at a density of 12,000 cells/well. After 20 h the supernatants were harvested after spinning the cell suspension at 450 × *g* and snap frozen. Medium without cells served as baseline. Cytokine production was measured with the Bio-Plex Pro Human Cytokine 27-plex Immunoassay (Bio-Rad) according the manufacturer's protocol.

SUPPLEMENTAL INFORMATION

Supplemental Information can be found online at https://doi.org/10.1016/j.stemcr.2019.12.010.

AUTHOR CONTRIBUTIONS

M.T. designed and performed experiments; A.C., H.B., H.P., E.d.W., M.C., and A.S.F. performed experiments; M.v.d.B. provided material and advice; and B.V., F.S., and R.H. provided advice. H.M. conceived, supervised, and performed experiments and wrote the manuscript with support from M.T., R.C.H., and F.S.

ACKNOWLEDGMENTS

The authors thank Daniela Salvatori and Tineke Kleinhoudt-Vliek for technical support, Karoly Szuhai (LUMC) for COBRA, and Janine Melsen (LUMC) for NK cell expertise. This study was supported by the LSBR foundation (H.M., H.B.) (LSBR09-11), the PON Foundation (A.C.), the European Commission (Marie Curie Individual Fellowship to M.T.), the Dutch Cancer Society (KWF) (H.J.P., M.T.), and Stichting VUmc CCA (M.T.).

Received: December 20, 2018
Revised: December 12, 2019
Accepted: December 17, 2019
Published: January 16, 2020

REFERENCES

Blom, B., Verschuren, M.C.M., Heemskerk, M.H.M., Bakker, A.Q., van Gastel-Mol, E.J., Wolvers-Tettero, I.L.M., van Dongen, J.J.M., and Spits, H. (1999). TCR gene rearrangements and expression of the pre-T cell receptor complex during human T-cell differentiation. Blood *93*, 3033–3043.

Brauer, P.M., Pessach, I.M., Clarke, E., Rowe, J.H., Ott de Bruin, L., Lee, Y.N., Dominguez-Brauer, C., Comeau, A.M., Awong, G., Felgentreff, K., et al. (2016). Modeling altered T-cell development with induced pluripotent stem cells from patients with RAG1-dependent immune deficiencies. Blood *128*, 783–793.

Bruggemann, M., van der Velden, V.H., Raff, T., Droese, J., Ritgen, M., Pott, C., Wijkhuijs, A.J., Gokbuget, N., Hoelzer, D., van Wering, E.R., et al. (2004). Rearranged T-cell receptor beta genes represent powerful targets for quantification of minimal residual disease in childhood and adult T-cell acute lymphoblastic leukemia. Leukemia *18*, 709–719.

Chang, C.W., Lai, Y.S., Westin, E., Khodadadi-Jamayran, A., Pawlik, K.M., Lamb, L.S., Jr., Goldman, F.D., and Townes, T.M. (2015). Modeling human severe combined immunodeficiency and correction by CRISPR/Cas9-Enhanced gene targeting. Cell Rep. *12*, 1668–1677.

Chen, X., Janssen, J.M., Liu, J., Maggio, I., t Jong, A.E.J., Mikkers, H.M.M., and Goncalves, M. (2017). In trans paired nicking triggers seamless genome editing without double-stranded DNA cutting. Nat. Commun. *8*, 657.

Clave, E., Araujo, I.L., Alanio, C., Patin, E., Bergstedt, J., Urrutia, A., Lopez-Lastra, S., Li, Y., Charbit, B., MacPherson, C.R., et al. (2018). Human thymopoiesis is influenced by a common genetic variant within the TCRA-TCRD locus. Sci. Transl. Med. *10*. https://doi.org/10.1126/scitranslmed.aao2966.

Dambrot, C., van de Pas, S., van Zijl, L., Brandl, B., Wang, J.W., Schalij, M.J., Hoeben, R.C., Atsma, D.E., Mikkers, H.M., Mummery, C.L., et al. (2013). Polycistronic lentivirus induced pluripotent stem cells from skin biopsies after long term storage, blood outgrowth endothelial cells and cells from milk teeth. Differentiation *85*, 101–109.

Dik, W.A., Pike-Overzet, K., Weerkamp, F., de Ridder, D., de Haas, E.F., Baert, M.R., van der Spek, P., Koster, E.E., Reinders, M.J., van Dongen, J.J., et al. (2005). New insights on human T cell development by quantitative T cell receptor gene rearrangement studies and gene expression profiling. J. Exp. Med. *201*, 1715–1723.

Ditadi, A., and Sturgeon, C.M. (2016). Directed differentiation of definitive hemogenic endothelium and hematopoietic progenitors from human pluripotent stem cells. Methods *101*, 65–72.

Dobbs, K., Tabellini, G., Calzoni, E., Patrizi, O., Martinez, P., Giliani, S.C., Moratto, D., Al-Herz, W., Cancrini, C., Cowan, M., et al. (2017). Natural killer cells from patients with recombinase-activating gene and non-homologous end joining gene defects comprise a higher frequency of CD56(bright) NKG2A(+++) cells, and yet display increased degranulation and higher perforin content. Front Immunol. *8*, 798.

Dvorak, C.C., and Cowan, M.J. (2010). Radiosensitive severe combined immunodeficiency disease. Immunol. Allergy Clin. North Am. *30*, 125–142.

Fischer, A. (2000). Severe combined immunodeficiencies (SCID). Clin. Exp. Immunol. *122*, 143–149.

Fronkova, E., Krejci, O., Kalina, T., Horvath, O., Trka, J., and Hrusak, O. (2005). Lymphoid differentiation pathways can be traced by TCR delta rearrangements. J. Immunol. *175*, 2495–2500.

Galic, Z., Kitchen, S.G., Subramanian, A., Bristol, G., Marsden, M.D., Balamurugan, A., Kacena, A., Yang, O., and Zack, J.A. (2009). Generation of T lineage cells from human embryonic stem cells in a feeder free system. Stem Cells *27*, 100–107.

Gaspar, H.B., Qasim, W., Davies, E.G., Rao, K., Amrolia, P.J., and Veys, P. (2013). How I treat severe combined immunodeficiency. Blood *122*, 3749–3758.

Gonçalves, M.A., Holkers, M., van Nierop, G.P., Wieringa, R., Pau, M.G., and de Vries, A.A.F. (2008). Targeted chromosomal insertion of large DNA into the human genome by a fiber-modified high-capacity adenovirus-based vector system. PLoS One *3*, e3084.

Hao, Q.L., George, A.A., Zhu, J., Barsky, L., Zielinska, E., Wang, X., Price, M., Ge, S., and Crooks, G.M. (2008). Human intrathymic lineage commitment is marked by differential CD7 expression: identification of CD7- lympho-myeloid thymic progenitors. Blood *111*, 1318–1326.

Kajiwara, M., Aoi, T., Okita, K., Takahashi, R., Inoue, H., Takayama, N., Endo, H., Eto, K., Toguchida, J., Uemoto, S., et al. (2012). Donor-dependent variations in hepatic differentiation from human-induced pluripotent stem cells. Proc. Natl. Acad. Sci. U S A *109*, 12538–12543.

Karo, J.M., Schatz, D.G., and Sun, J.C. (2014). The RAG recombinase dictates functional heterogeneity and cellular fitness in natural killer cells. Cell *159*, 94–107.

Kilpinen, H., Goncalves, A., Leha, A., Afzal, V., Alasoo, K., Ashford, S., Bala, S., Bensaddek, D., Casale, F.P., Culley, O.J., et al. (2017). Common genetic variation drives molecular heterogeneity in human iPSCs. Nature *546*, 370–375.

Laskowski, T.J., Van Caeneghem, Y., Pourebrahim, R., Ma, C., Ni, Z., Garate, Z., Crane, A.M., Li, X.S., Liao, W., Gonzalez-Garay, M., et al. (2016). Gene correction of iPSCs from a Wiskott-Aldrich syndrome patient normalizes the lymphoid developmental and functional defects. Stem Cell Reports *7*, 139–148.

Lertkiatmongkol, P., Liao, D., Mei, H., Hu, Y., and Newman, P.J. (2016). Endothelial functions of platelet/endothelial cell adhesion molecule-1 (CD31). Curr. Opin. Hematol. *23*, 253–259.

Ma, Y., Pannicke, U., Schwarz, K., and Lieber, M.R. (2002). Hairpin opening and overhang processing by an Artemis/DNA-dependent protein kinase complex in nonhomologous end joining and V(D)J recombination. Cell *108*, 781–794.

Menon, T., Firth, A.L., Scripture-Adams, D.D., Galic, Z., Qualls, S.J., Gilmore, W.B., Ke, E., Singer, O., Anderson, L.S., Bornzin, A.R., et al. (2015). Lymphoid regeneration from gene-corrected SCID-X1 subject-derived iPSCs. Cell Stem Cell *16*, 367–372.

Michaud, A., Dardari, R., Charrier, E., Cordeiro, P., Herblot, S., and Duval, M. (2010). IL-7 enhances survival of human CD56bright NK cells. J. Immunother. *33*, 382–390.

Milush, J.M., Long, B.R., Snyder-Cappione, J.E., Cappione, A.J., 3rd, York, V.A., Ndhlovu, L.C., Lanier, L.L., Michaelsson, J., and Nixon, D.F. (2009). Functionally distinct subsets of human NK cells and monocyte/DC-like cells identified by coexpression of CD56, CD7, and CD4. Blood *114*, 4823–4831.

Narni-Mancinelli, E., Vivier, E., and Kerdiles, Y.M. (2011). The 'T-cell-ness' of NK cells: unexpected similarities between NK cells and T cells. Int. Immunol. *23*, 427–431.

Notarangelo, L.D., Kim, M.S., Walter, J.E., and Lee, Y.N. (2016). Human RAG mutations: biochemistry and clinical implications. Nat. Rev. Immunol. *16*, 234–246.

Shinkai, Y., Rathbun, G., Lam, K.P., Oltz, E.M., Stewart, V., Mendelsohn, M., Charron, J., Datta, M., Young, F., Stall, A.M., et al. (1992). RAG-2-deficient mice lack mature lymphocytes owing to inability to initiate V(D)J rearrangement. Cell *68*, 855–867.

Six, E.M., Benjelloun, F., Garrigue, A., Bonhomme, D., Morillon, E., Rouiller, J., Cacavelli, L., Blondeau, J., Beldjord, K., Hacein-Bey-Abina, S., et al. (2011). Cytokines and culture medium have a major impact on human in vitro T-cell differentiation. Blood Cells Mol Dis *47*, 72–78.

Takahashi, K., Tanabe, K., Ohnuki, M., Narita, M., Ichisaka, T., Tomoda, K., and Yamanaka, S. (2007). Induction of pluripotent stem cells from adult human fibroblasts by defined factors. Cell *131*, 861–872.

Themeli, M., Kloss, C.C., Ciriello, G., Fedorov, V.D., Perna, F., Gonen, M., and Sadelain, M. (2013). Generation of tumor-targeted human T lymphocytes from induced pluripotent stem cells for cancer therapy. Nat. Biotechnol. *31*, 928–933.

Timmermans, F., Velghe, I., Vanwalleghem, L., De Smedt, M., Van Coppernolle, S., Taghon, T., Moore, H.D., Leclercq, G., Langerak, A.W., Kerre, T., et al. (2009). Generation of T cells from human embryonic stem cell-derived hematopoietic zones. J. Immunol. *182*, 6879–6888.

Vodyanik, M.A., Bork, J.A., Thomson, J.A., and Slukvin, , II.. (2005). Human embryonic stem cell-derived CD34+ cells: efficient production in the coculture with OP9 stromal cells and analysis of lymphohematopoietic potential. Blood *105*, 617–626.

Vosshenrich, C.A., Garcia-Ojeda, M.E., Samson-Villeger, S.I., Pasqualetto, V., Enault, L., Richard-Le Goff, O., Corcuff, E., Guy-Grand, D., Rocha, B., Cumano, A., et al. (2006). A thymic pathway of mouse natural killer cell development characterized by expression of GATA-3 and CD127. Nat. Immunol. *7*, 1217–1224.

Warlich, E., Kuehle, J., Cantz, T., Brugman, M.H., Maetzig, T., Galla, M., Filipczyk, A.A., Halle, S., Klump, H., Scholer, H.R., et al. (2011). Lentiviral vector design and imaging approaches to visualize the early stages of cellular reprogramming. Mol. Ther. *19*, 782–789.

Weerkamp, F., Baert, M.R., Brugman, M.H., Dik, W.A., de Haas, E.F., Visser, T.P., de Groot, C.J., Wagemaker, G., van Dongen, J.J., and Staal, F.J. (2006). Human thymus contains multipotent progenitors with T/B lymphoid, myeloid, and erythroid lineage potential. Blood *107*, 3131–3137.

Wiekmeijer, A.S., Pike-Overzet, K., IJspeert, H., Brugman, M.H., Wolvers-Tettero, I.L., Lankester, A.C., Bredius, R.G., van Dongen, J.J., Fibbe, W.E., et al. (2016). Identification of checkpoints in human T-cell development using severe combined immunodeficiency stem cells. J. Allergy Clin. Immunol. *137*, 517–526.e3.

Yu, J., Freud, A.G., and Caligiuri, M.A. (2013). Location and cellular stages of natural killer cell development. Trends Immunol. *34*, 573–582.

Yu, J., Vodyanik, M.A., Smuga-Otto, K., Antosiewicz-Bourget, J., Frane, J.L., Tian, S., Nie, J., Jonsdottir, G.A., Ruotti, V., Stewart, R., et al. (2007). Induced pluripotent stem cell lines derived from human somatic cells. Science *318*, 1917–1920.

Zeng, J., Tang, S.Y., Toh, L.L., and Wang, S. (2017). Generation of "Off-the-Shelf" natural killer cells from peripheral blood cell-derived induced pluripotent stem cells. Stem Cell Reports *9*, 1796–1812.

Stem Cell Reports, Volume *14*

Supplemental Information

iPSC-Based Modeling of *RAG2* Severe Combined Immunodeficiency Reveals Multiple T Cell Developmental Arrests

Maria Themeli, Amiet Chhatta, Hester Boersma, Henk Jan Prins, Martijn Cordes, Edwin de Wilt, Aïda Shahrabi Farahani, Bart Vandekerckhove, Mirjam van der Burg, Rob C. Hoeben, Frank J.T. Staal, and Harald M.M. Mikkers

Inventory of Supplemental Information

Themeli et al.

Figure S1: iPSC characterization data that are not presented in Figure 1.

Figure S2: Hematopoietic differentiation data that are not incorporated into Figure 2.

Figure S3: T cell differentiation data of all (3 *RAG2SCID* and 2 *RAGC*) clones analysed.

Figure S4: TCR rearrangement data additional to the results shown in Figure 4.

Figure S5: Expression analysis of monocytic, DC, and NK cell markers in the CD56+ populations from *RAG2SCID* and *RAG2C* that are not presented in Figure 5. In addition, the marker profile of the CD56+ cells from umbilical cord blood that served as positive control in the stimulation experiment is depicted.

Figure S1

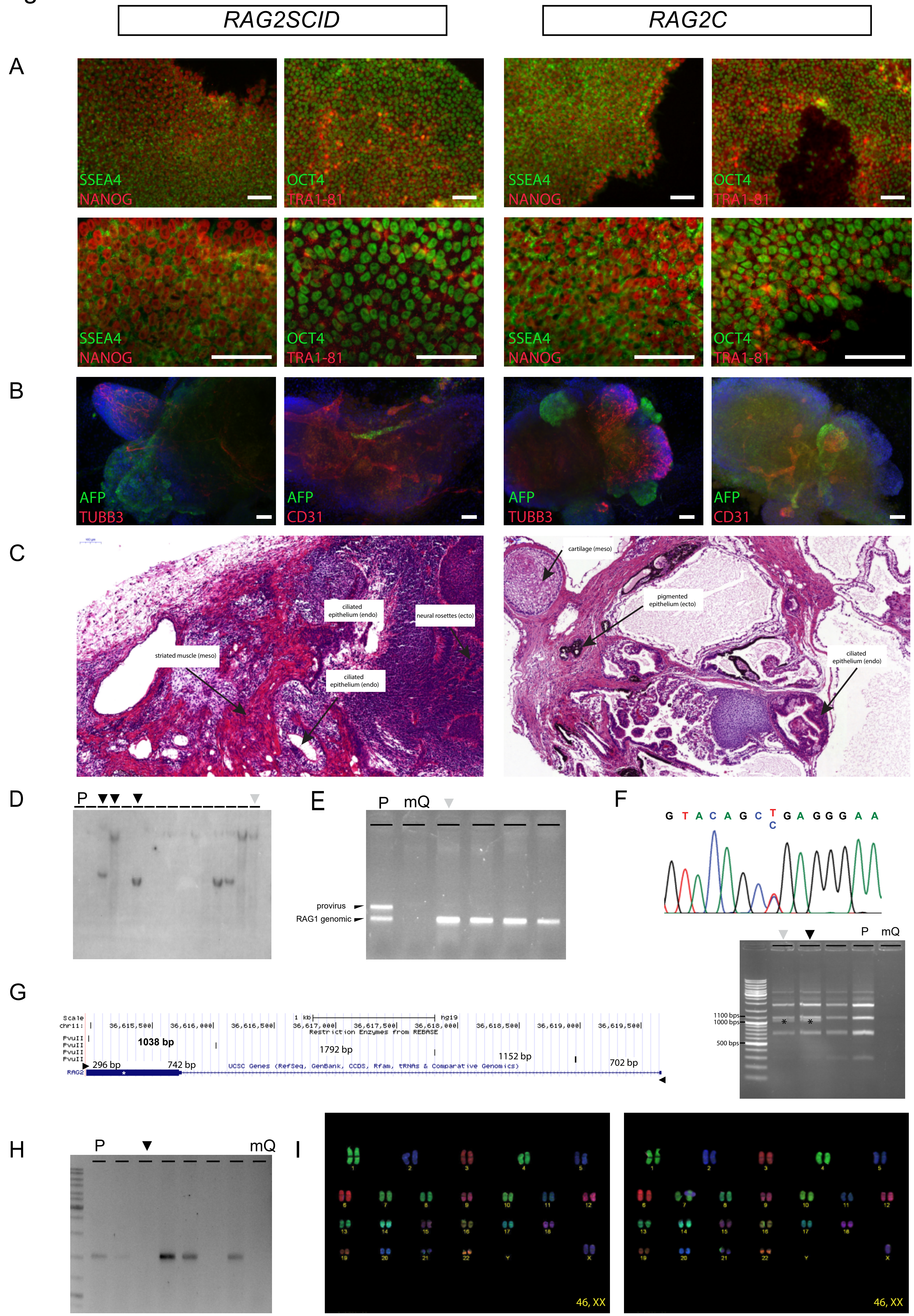
RAG2SCID
RAG2C
A
SSEA4
NANOG
OCT4
TRA1-81
B
AFP
TUBB3
CD31
C
striated muscle (meso)
ciliated epithelium (endo)
neural rosettes (ecto)
cartilage (meso)
pigmented epithelium (ecto)
D
P
E
P mQ
provirus
RAG1 genomic
F
G T A C A G C T G A G G G A A
C
P mQ
1100 bps
1000 bps
500 bps
G
1038 bp
1792 bp
1152 bp
296 bp
742 bp
702 bp
RAG2
H
P
mQ
I
46, XX
46, XX

Figure S1. Characterization of generated *RAG2SCID* and repaired *RAG2C* iPSC. A. Immunofluorescence of the pluripotent stem cell markers SSEA4, NANOG, OCT4 and TRA1-81. B. Immunofluorescence of the spontaneous differentiation into three germ layers in vitro. AFP (endoderm), TUBB3 (ectoderm), CD31 (mesoderm). C. HE staining of teratomas. Structures of the three germ layers are indicated by arrows. D. Provirus insertion analysis of multiple *RAG2SCID* clones. Arrows indicate clones used in the differentiation experiments. E. PCR to assess removal of the provirus from one *RAG2SCID* clone (gray arrow in D) by Fib50.FLPe treatment. F. DNA sequencing chromatogram of *RAG2C2* indicating repair of the nonsense mutation in one of the mutant alleles. G. UCSC genome browser (hg19) view depicting the PvuII fragments of the amplified *RAG2* region (left). The nonsense mutation (*) in *RAG2* creates an additional PvuII restriction site in the 1038 bps fragment yielding one 296 bps and one 742 bps fragment. PvuII restriction of the amplified *RAG2* fragment shows an extra 1038 bps fragment (indicated by black asterisk) in the repaired clones (*RAG2C1* (grey arrow) and *RAG2C2* (black arrow). P= one of the parental *RAG2SCID* clones. H. PCR analysis of the selection cassette. Black arrow represents clone *RAG2C1* from which the selection cassette was removed. P= the parental clone. H. Karyotype analysis using COBRA-FISH. *RAG2SCID* (left), *RAG2C* (right). One of the X-chromosomes (*RAG2C*) is located on top of one of the chromosomes 7 in this spread. Scale bars represent 100 μM.

Figure S2

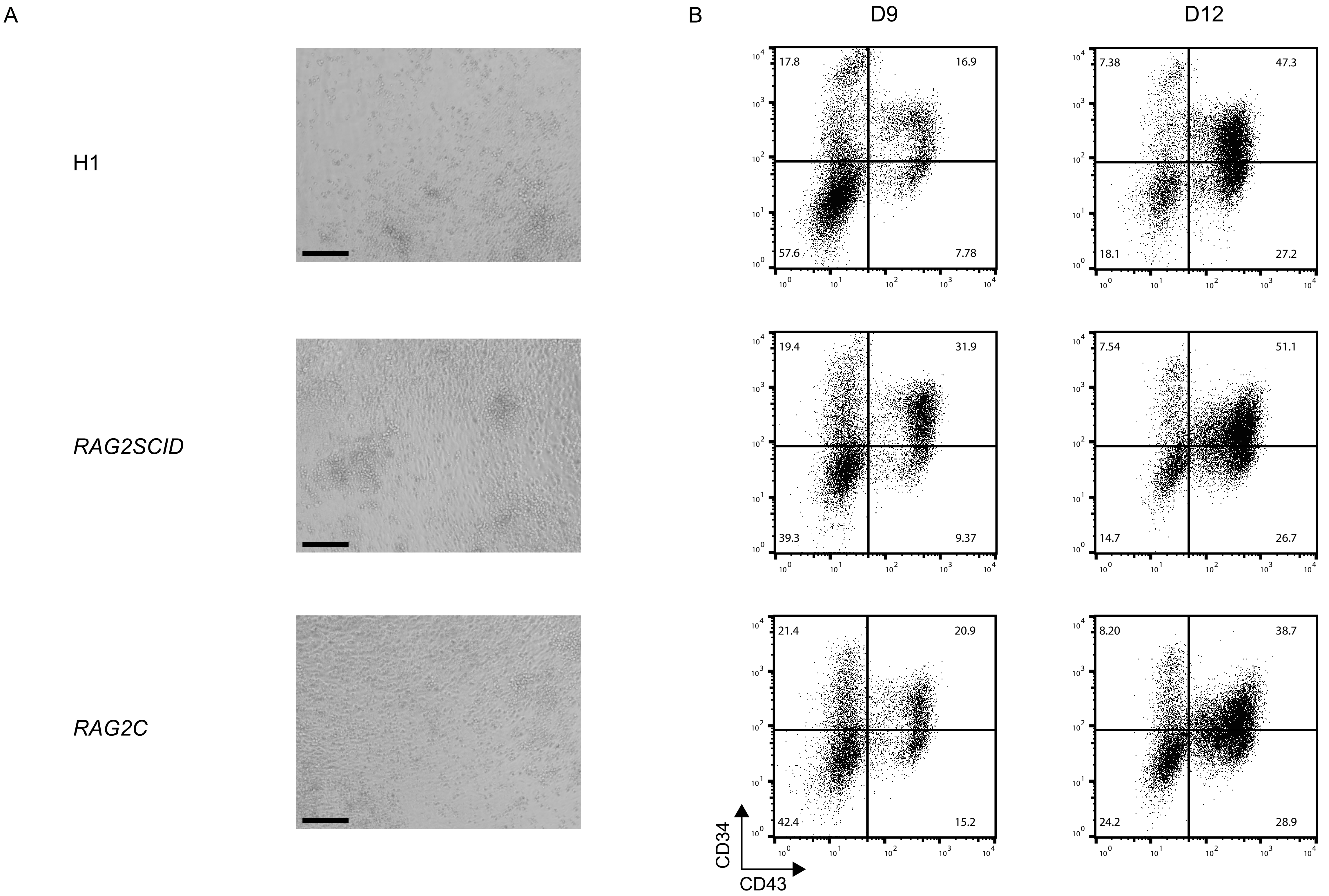

Figure S2. Early hematopoietic differentiation RAG2SCID and repaired RAG2C iPSC is very similar. A. Bright field images of hematopoietic differentiation cultures at day 9 of differentiation. B. Flow cytometric analysis of CD34 and CD43 in hematopoietic differentiation cultures at day 9 (left) and day 12 (right) of differentiation. H1 (upper panel), *RAG2SCID* (middle panel), and *RAG2C* (lower panel). Scale bar represents 200 μM. Plots are a representative example from three independent experiments.

Figure S3

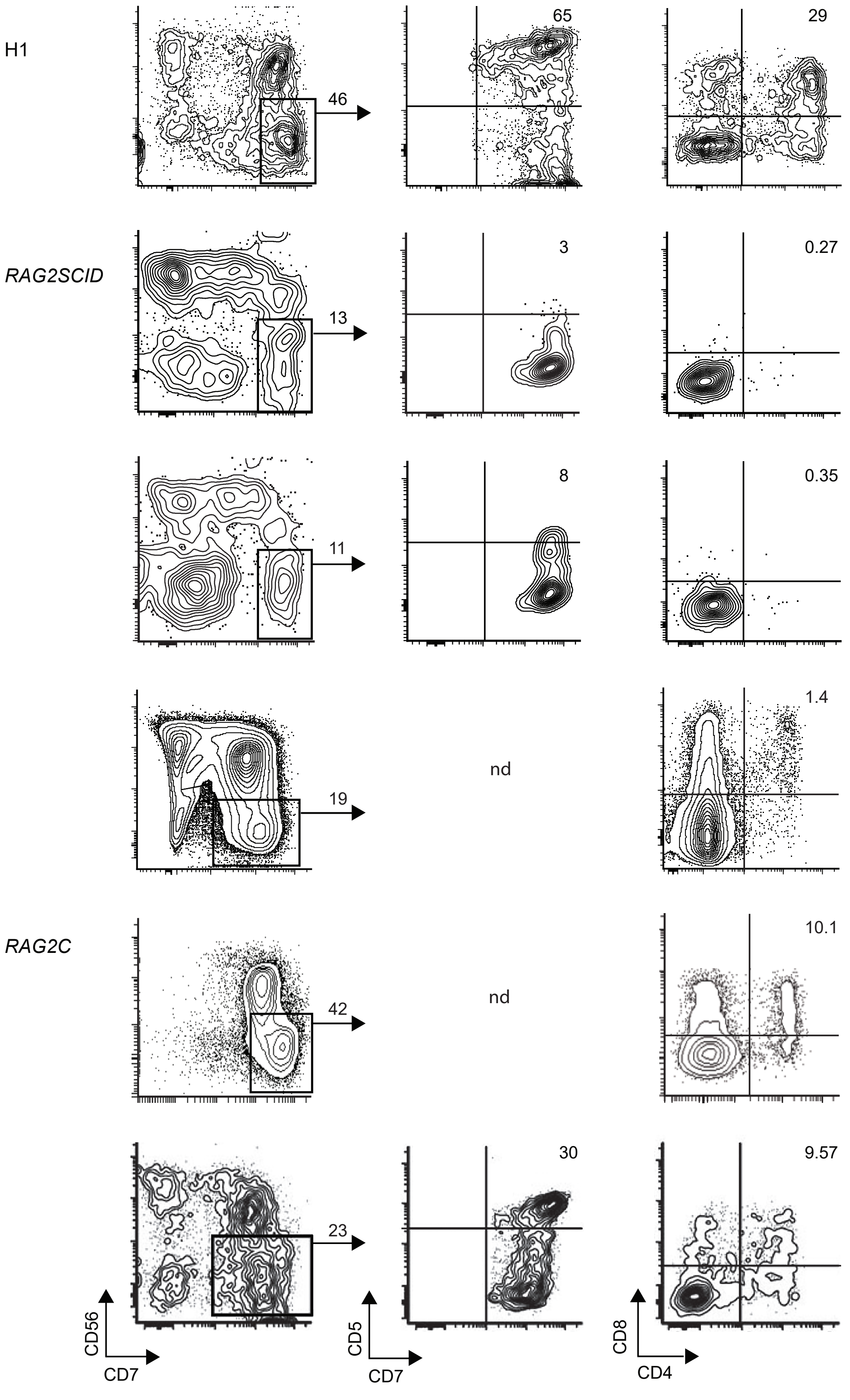

Figure S3. T cell differentiation and rescue across multiple mutant and repaired RAG2SCID iPSC clones. Additional representative flow cytometry plots showing the differentiation into the T lineage of H1 ESCs, three *RAG2SCID* iPSC clones and two repaired isogenic *RAG2C* iPSC clones. nd = not done

Figure S4

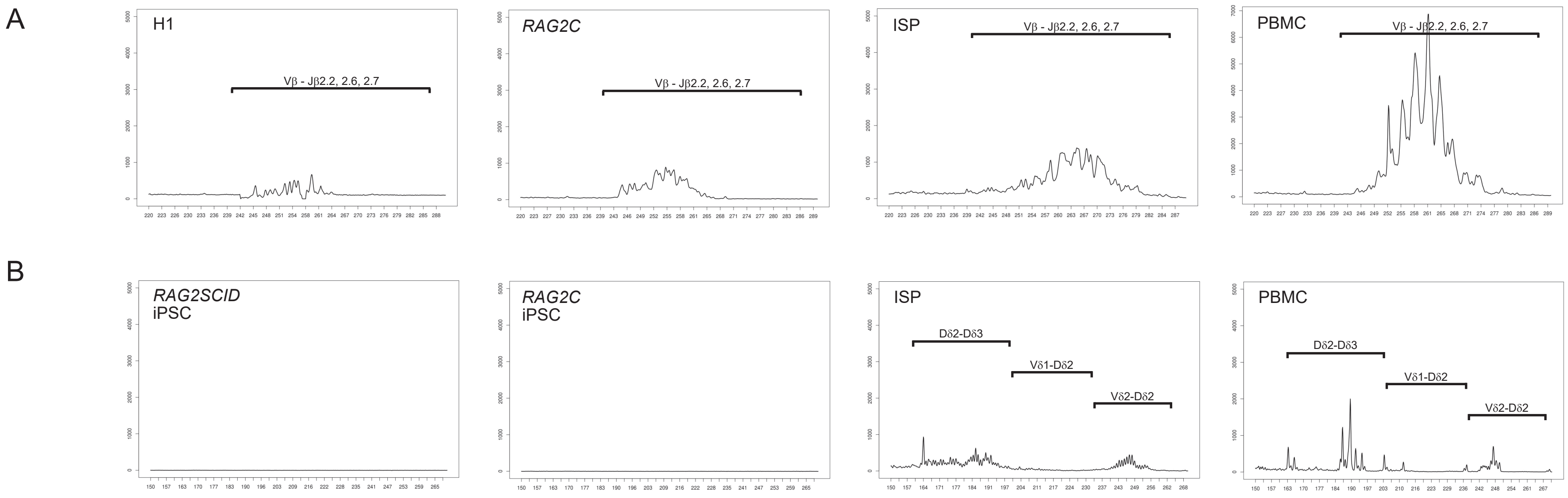

Figure S4. TCR rearrangements in repaired RAG2SCID T cells.

A. GeneScan analysis of TCB (Vβ-Jβ2) rearrangements in CD4+CD8+ sorted H1 and RAG2C cells, as well as in primary human ISP cells and peripheral blood mononuclear cells (PBMC). B. GeneScan rearrangements of early TCD rearrangements in RAG2SCID and RAG2C iPSC, primary ISP cells and PBMC.

Figure S5

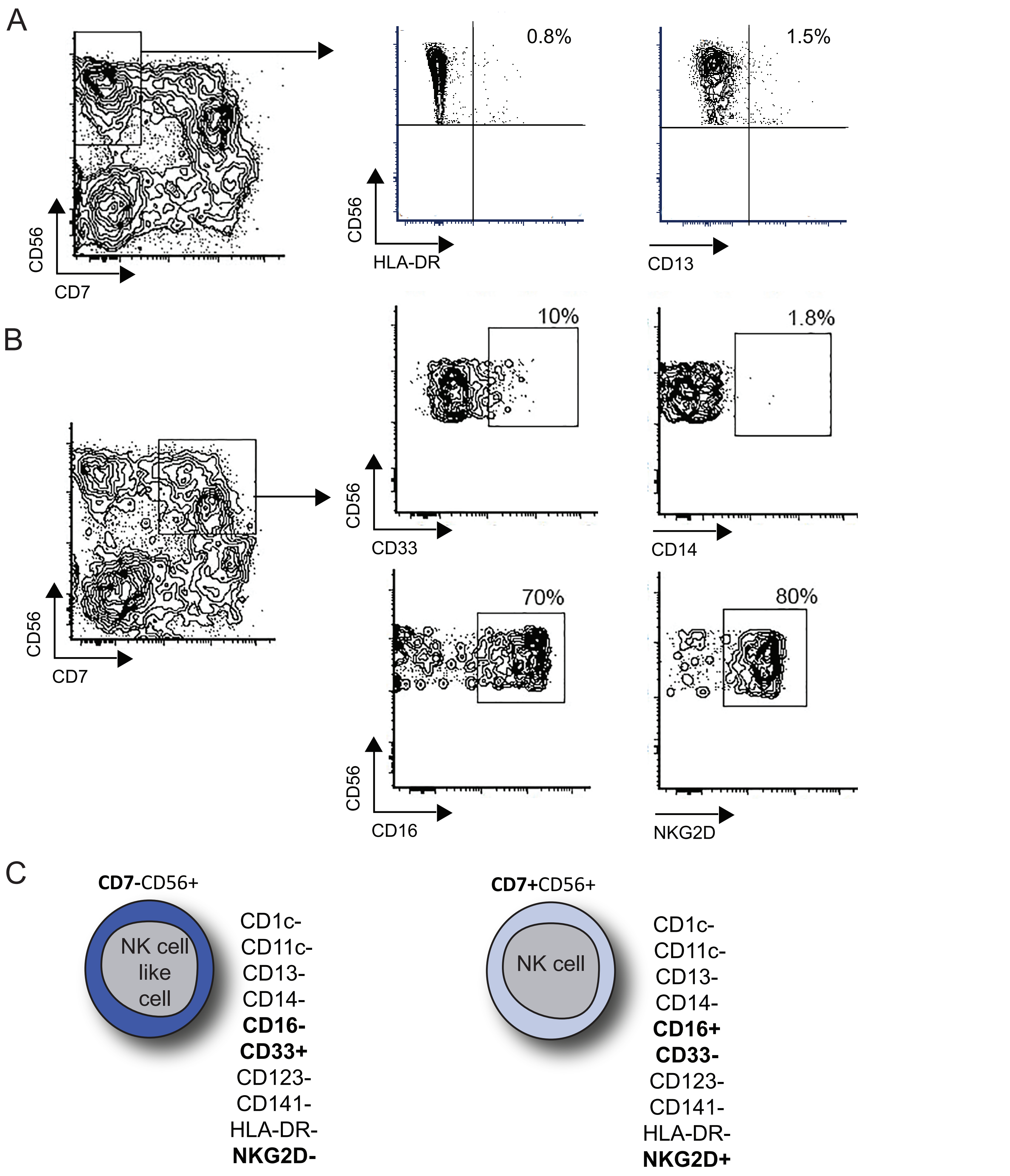

Figure S5. Characterization of *RAG2SCID* iPSC-derived CD7-CD56+ and CD7+CD56+.
A. Expression of HLA-DR and CD13 in CD7-CD56+ cells and B. Expression of myeloid (CD14 and CD33) and NK cell markers (CD16 and NKG2D) in CD7+CD56+ cells. C. Schematic overview of the tested marker profile of *RAG2SCID* iPSC-derived CD7-CD56+ and CD7+CD56+ cells. Differences are indicated in bold.

Table S1. Oligo sequences.

Primer name	Purpose	Sequence 5' to 3'	
coKLF4SOX2for	Provirus detection	GAAGCGACGAGCTGACCCGG	
coKLF4SOX2rev	Provirus detection	TGCAGGGCTCTCAGCCGCTT	
REChRAGMCSupper	RAG recombineering site in pSuperCOS	AAGAATTCGAA**GGCCGGTGGGGACAGGGCTGAGCCAGCACCAACCACTCAGCCTTTGAGA**TATCTA **GACTAACACACTGCCAAAATGTGGCAT**GAATTCAAA	11: 36590857-36590905<br><br>11:36619551-36619577
REChRAGMCSlower	RAG recombineering site in pSuperCOS	TTTGAATTC**ATGCCACATTTTGGCAGTGTGTTAGTCC**TAGATA **TCTCAAAGGCTGAGTGGTTGGTGCTGGCTCAGCCCTGTCCCCACCGGCC**TTCGAATTCTT	
SbfILoxPfor	Creating SbfI sites 5' and 3' of LoxpPGKPurΔTKLoxP	CCTGCAGGACCCTTAATATAACTTCGTATAATGTATG	
SbfILoxPrev	Creating SbfI sites 5' and 3' of LoxpPGKPurΔTKLoxP	CCTGCAGGACCTAATAACTTCGTATAGCATAC	
hRAG2For	HDR analysis	GCAAGACTGTGCAATTCACAGCTGG	11: 36614962-36614986
hRAG2Rev	HDR analysis	CCCTCTGGCCTTCAGGTAGGTCTG	11: 36619643-36619666
hRAG1For_nested	gDNA control	GCAAAGAGGTTCCGCTATGATTCAGC	11: 36596523-36596548
hRAG1Rev	gDNA control	GGCTTGCAACACAGTTCAGAGTTAGG	11: 36596799-36596824
PGKrev	PuΔTK excision analysis	CTTGGCTGGACGTAAACTCCTCTTC	
hRAGPuTKexcFor	PuΔTK excision analysis	CCATCCCACAGCTCCACTAGGCA	11: 36602877-36602899
hRAGPuTKexcRev	PuΔTK excision analysis	CTAACTTGCACAGCCTTTGGAC	11: 36603216-36603237
Vδ1	TCD rearrangements	ATGCAAAAAGTGGTCGCTATT	
Vδ2	TCD rearrangements	ATACCGAGAAAAGGACATCTATG	
Dδ2-5'	TCD rearrangements	AGCGGGTGGTGATGGCAAAGT	
Dδ3-3' (HEX)	TCD rearrangements	TATAGGAGTGGGACCCAGGGT	
